# Twelve new and exciting Annonaceae from the Neotropics

**DOI:** 10.3897/phytokeys.126.33913

**Published:** 2019-07-02

**Authors:** Paul J.M. Maas, Lubbert Y.Th. Westra, Lars W. Chatrou, Nadja Verspagen, Heimo Rainer, Nelson A. Zamora, Roy H.J. Erkens

**Affiliations:** 1 Naturalis Biodiversity Center, section Botany, P.O. Box 9517, 2300 RA Leiden, The Netherlands Naturalis Biodiversity Center Leiden Netherlands; 2 Ghent University, Systematics and Evolutionary Botany lab., K.L. Ledeganckstraat 35, 9000 Ghent, Belgium Ghent University Gent Belgium; 3 Maastricht Science Programme, Maastricht University, P.O.Box 616, 6200 MD, Maastricht, The Netherlands Maastricht University Maastricht Netherlands; 4 Herbarium, Faculty Center Botany, Department of Plant Systematics and Evolution, Faculty of Life Sciences, Universität Wien, Rennweg 14, Wien A-1030, Austria Universität Wien Wien Austria; 5 Herbario Nacional de Costa Rica, Departemento de Historia Natural, Museo Nacional de Costa Rica. Apartado 749-1000, San José, Costa Rica Museo Nacional de Costa Rica San José Costa Rica

**Keywords:** Annonaceae, *
Annona
*, *
Guatteria
*, *
Klarobelia
*, *
Tetrameranthus
*, *
Xylopia
*, neotropics, new species, taxonomy

## Abstract

As a result of concerted efforts of the community of Annonaceae taxonomists, increasingly detailed knowledge of the diversity of the Neotropical genera has been documented. With the exception of just two large genera, *Annona* and *Xylopia*, all Neotropical Annonaceae have been revised within the last 25 years. Subsequent to these publications, many new specimens have been collected and sent to us in Leiden for identification. These included a number that, despite the advanced state of taxonomic knowledge, proved to represent rarely collected, undescribed species. Here we describe 12 new species of *Annona*, *Guatteria*, *Klarobelia*, *Tetrameranthus*, and *Xylopia*. These species serve to illustrate the still underestimated diversity of the Neotropical flora, even in well studied plant groups like Annonaceae.

## Introduction

The Neotropics are rich in plant diversity but how rich they are is still a matter of serious debate. For instance, estimating how many tree species exist in the Amazon basin is a non-trivial exercise (e.g. ter Steege et al. 2016, Cardoso et al. 2017, ter Steege et al. 2019). Yet, a proper estimate of this diversity is essential in order to answer questions on the ecology, evolution and origins of the Neotropical flora and fauna and the processes that are responsible for creating and maintaining its hyperdiverse communities (Cardoso et al. 2017).

Annonaceae is a family of ca. 2450 species of trees and lianas that is well represented in the Neotropical tree flora with ca. 950 species (Rainer and Chatrou 2006, Maas et al. 2011). Understanding the species delineations in this family therefore contributes noticeably to the aim of understanding Neotropical tree diversity. Ongoing revisional work on Neotropical Annonaceae has already led to the revision of almost all genera during the last 25 years (for an overview see Erkens et al. 2017). Still, in this article new species in several genera of Annonaceae are described, and some notes are added on congeneric species of which the circumscription is affected by the newly described species.

The genus *Annona* L. is distributed in tropical America and tropical Africa. It is the second-largest genus of Annonaceae in the Neotropics with a total number of ca. 160 species, four of which inhabit Africa. Nowadays, it is taken in its original concept including two genera which were treated for a long time as segregates. This concerns *Raimondia* Saff. and *Rollinia* A.St.-Hil.: see the comments under the new species *Annonacaput-medusae* and *A.oleifolia*.

The new species of *Klarobelia* Chatrou is an addition to the revision of Chatrou (1998). The Neotropical distribution of this genus is similar to that of *Mosannona* and *Cremastosperma* (Pirie et al. 2018). As in *Mosannona*, species of *Klarobelia* have small, non-overlapping distributions. Species have been discovered before (e.g. *K.megalocarpa*, Chatrou 1998) when new areas, in between known distribution areas, were first disclosed by plant collectors. The new species described has become known through collecting efforts, especially in the Peruvian province of Oxapampa.

*Guatteria* Ruiz & Pav. is the largest Neotropical genus of Annonaceae with more than 175 species. It is distributed from Mexico to south-eastern Brazil and was recently revised by Maas et al. (2015). Both because of very recently received specimens and a reinterpretation of some species complexes, several new species of *Guatteria* have to be described in the present paper.

The very small and poorly collected genus *Tetrameranthus* R.E.Fr., quite aberrant from all other genera of Annonaceae because of its spirally arranged (instead of distichous) leaves, was treated twice by Westra (Westra 1985; Westra and Maas 2012). *Tetrameranthus* is a small genus with 8 species, occurring in the Amazon Region, neighbouring French Guiana and the Colombian state of Chocó. Recently we received very rich flowering and fruiting material, accompanied by nice field photographs of an undescribed species from Amazonian Peru which is herewith described.

The genus *Xylopia* L., the only genus of Annonaceae occurring in three continents, has recently been revised for Africa (Johnson and Murray 2018), but treatments for the Asian and American species are needed. The last revision of the Neotropical species of *Xylopia* dates back to Fries (Fries 1930, various supplements). A recent estimation by David Johnson of the total number of species of *Xylopia* in the tropics is 160, whereas ca. 50 species inhabit the Neotropics (DM Johnson pers. comm.). In the present paper a new Colombian species with very distinctive leaf features is described.

## Materials and methods

All IUCN Redlist assessments were done on data from herbarium collections and following the IUCN guidelines (IUCN 2012, 2017). Only criterion B could be used for the assessments since data on species’ populations (Criteria A, C and D) and extinction probability (Criterion E) were lacking. The area of occupancy (AOO) was calculated by overlaying the occurrence data points with a 2×2 km grid and adding the area of all occupied cells. The extent of occurrence (EOO) was determined by calculating the area of the minimum convex polygon that was drawn around the outer occurrence points. Both AOO and EOO were calculated in R using the ConR package (Dauby et al. 2017). For several species only one to a few data points were available and these were considered Data Deficient. Although assessments can still be carried out for species with such low numbers of collections (Rivers et al 2011) it was unclear whether a lack of data caused the apparent rarity of these species or if they were actually rare, since no other data was available to the authors. It is important to note that a species with such small amounts of data can be endangered and thus a reassessment is needed when more data becomes available.

For those species that were not considered Data Deficient (i.e. had more than 3 collections) data on forest cover loss (Hansen et al. 2013) was investigated to infer if habitat loss was a threat for those species. This was the case if the species occurred in regions where forest cover loss had been observed in the past years. For this assessment, it was assumed that forest cover loss was regulated differently outside and across different protected areas, and thus every occurrence point within a particular protected area was considered as one location. For occurrence points that were not situated in a protected area, a 10 × 10 km grid was used to estimate separate locations.

It must be noted that no extensive survey on the occurrence of these species was undertaken; the AOO and number of locations are therefore a conservative estimate.

## Taxonomy

### Annona caput-medusae

Taxon classificationPlantaeMagnolialesAnnonaceae

Westra & H.Rainer sp. nov.

urn:lsid:ipni.org:names:77199050-1

Figs 1 , 2

#### Diagnosis.

*Annonacaput-medusae* resembles cauliflorous specimens of *A.quinduensis* Kunth (formerly *Raimondiaquinduensis*), but differs by the shorter pedicels (7–11 vs. 10–30 mm long) and smaller seeds (ca. 6 vs. 10–14 mm long).

#### Type.

COLOMBIA, Antioquia: Mun. Anorí, electric power plant, road to Aljibes, 7°19'61"N, 75°02'407"W, 350 m, 26 Mar 1996 (fl), *Fonnegra et al. 5935* (holotype: HUA! [HUA104142]; isotype: MO! [MO1958355]).

#### Description.

*Tree* 5–7 m tall, cauliflorous; young twigs rather densely covered with appressed brown hairs < 0.5 mm long, soon glabrous. *Leaves*: petioles 16–18 by 2 mm; lamina narrowly elliptic, 28–30 by 9–12 cm (leaf index 2.5–3.1), membranous, greenish grey above *in sicco*, somewhat lighter so below, glabrous above except for the large veins sparsely covered with erect, brown hairs, sparsely covered with appressed hairs to glabrous below, base obtuse, extreme base very shortly attenuate, apex acuminate (acumen 10–15 mm long), primary vein impressed to flat above, secondary veins ca. 15, not loop-forming or loop-forming close to the apex (shortest distance between loops and margin ca. 2 mm), tertiary veins mostly percurrent, domatia present in axils of part of the secondary veins; plants androdioecious, probably: only bisexual flowers seen. *Inflorescence* borne on the stem on older branches, much-branched thyrsoids bearing many flowers in succession; pedicels 7–11 by 1–2 mm, gradually widening from base to flower, densely covered with appressed, brown hairs to 0.2 mm long; bracts triangular-ovate or broadly triangular-ovate, outer side densely covered with hairs 0.1–0.2 mm long, more or less persistent, upper bract 0.3–0.4 mm from base of pedicel; flower buds narrowly conical; sepals free or connate at the base, broadly ovate to triangular-ovate, ca. 1 mm long, appressed, later spreading to reflexed, apex acuminate, outer side densely covered with brown hairs; outer petals connate at the base, narrowly triangular, ca. 20 by 5 mm, outer side densely covered with brown hairs, inner petals ca. 0.4 the length of the outer ones, torus ca. 3 mm long, the lower third beset with stamens, the apical two-thirds beset with carpels; stamens ca. 150, ca. 1.5 mm long, anther oblong, ca. 1 mm long, no apical prolongation of connective; carpels 150–200. *Fruit* ellipsoid, ca. 6.5 by 3.5 cm, densely covered with brown hairs ca. 0.2 mm long in young stage, becoming glabrous, areoles not or weakly protruding, not apiculate. *Seeds* ca. 6 by 5 mm, brown.

**Figure 1.**
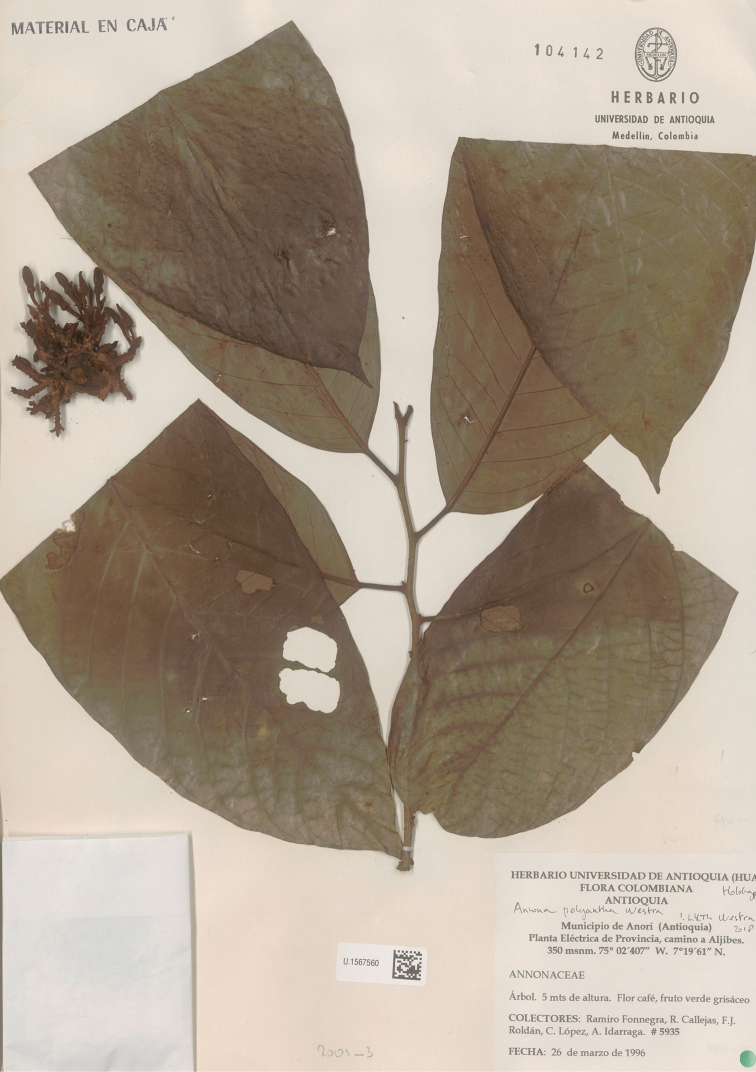
*Annonacaput-medusae* Westra & H.Rainer. Flowering specimen (*Fonnegra et al. 5935*, holotype HUA).

**Figure 2.**
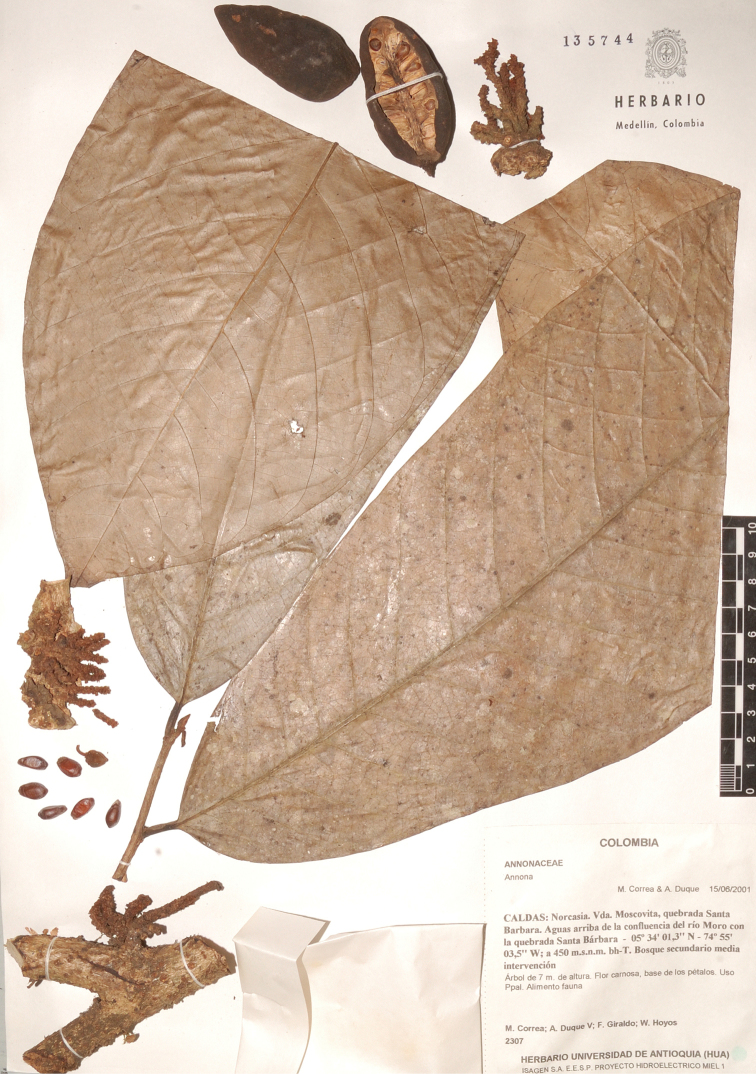
*Annonacaput-medusae* Westra & H.Rainer. Fruiting specimen (*Correa et al. 2307*, HUA).

#### Distribution.

**Figure 3.**
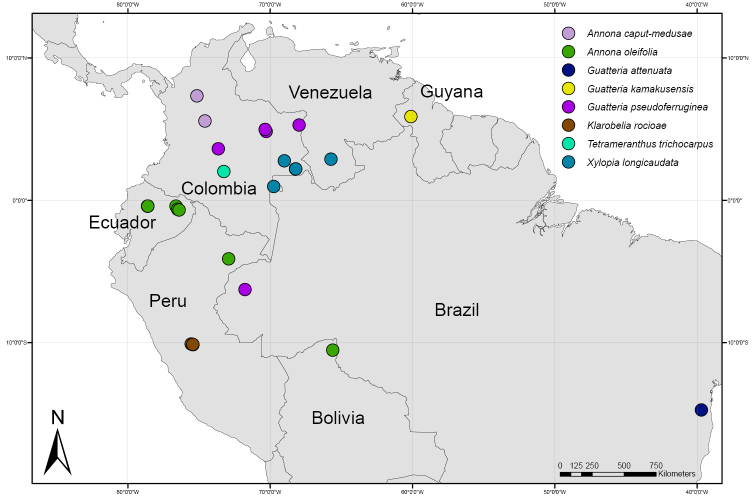
Distribution map of *Annonacaput-medusae*, *A.oleifolia*, *Guatteriaattenuata*, *G.kamakusensis*, *G.pseudoferruginea*, *Klarobeliarocioae*, and *Xylopialongicaudata*.

Colombia (Antioquia, Caldas) (Fig. 3).

#### Habitat and ecology.

On industrial ground, in secondary forest. At elevations of 350–450 m. Flowering: March; fruiting: June.

#### Notes.

A domatium here is a small thin membrane in the axil spanning the distance between primary vein and secondary vein. It conforms to the *Annonamuricata* type (Van den Bos et al. 1989).

*Annonacaput-medusae* clearly falls within a distinct group formerly known as the segregate genus *Raimondia* (Safford 1913; Westra 1995), but (re-)united later with *Annona* (Rainer 2007). When using Westra’s key *A.caput-medusae* comes closest to *A.quinduensis* Kunth which generally is not cauliflorous. Whereas *A.quinduensis* normally is found at higher elevations up to 2500 m, *A.caput-medusae*, as known from the scanty material collected thus far, occurs at elevations below 500 m. The flowers we examined appear to be bisexual. However, given the obvious similarity with other former *Raimondia* species, which are all androdioecious, staminate flowers might be expected in *A.caput-medusae* as well.

#### Etymology.

Caput (L) = head. Medusa, an ancient Greek goddess whose head was covered with snakes. Referring to the shape of the inflorescence.

#### Preliminary IUCN conservation status.

DD. This species is only known from two localities. Although the collections are not made near each other, more data are needed to determine the AOO and EOO. Also, the current population size and population trend of this species are unknown. Habitat loss because of forest cover loss is a possible threat for this species of *Annona* given its occurrence in fragmented forest areas. However, since proper data on the distribution of this taxon is lacking, we assessed it as Data Deficient.

#### Other specimen examined.

**COLOMBIA. Caldas**: Norcasia, Vereda Moscovita, quebrada Santa Bárbara, 5°34'N, 74°35'W, 450 m, 15 Jun 2001 (fr), *Correa et al. 2307* (HUA).

### Annona oleifolia

Taxon classificationPlantaeMagnolialesAnnonaceae

Westra & H.Rainer sp. nov.

urn:lsid:ipni.org:names:77199051-1

Figs 4 , 5

#### Diagnosis.

When using the key of the Flora Neotropica Monograph of *Rollinia* (Maas, Westra et al. 1992) *Annonaoleifolia* keys out to the SE Brazilian *Annonaneosericea* H.Rainer by an indument of appressed hairs on the lower side of the lamina, non-gibbous sepals, and narrow wings, but it is very distinct from that species by the very low number of carpels (≤25 vs. ≥100, respectively), very narrow leaves (leaf index ≥5 vs. 2.5–3) and the slightly recurved instead of horizontal to erect wings in *Annonaneosericea*.

#### Type.

ECUADOR, Napo: La Joya de los Sachas, Parroquia Pompeya, Campamento de Maxus, Carretera Maxus km 1–4, 00°25'S, 78°36'W, 235 m, 10–18 Aug 1993 (fr), *Grijalva et al. 637* (holotype: QCNE! [QCNE75007]; isotype: U! [U1567540]).

#### Description.

*Small tree* 2–3 m tall, to 3.5 cm diam.; young twigs rather densely to sparsely covered with appressed, brownish hairs < 0.5 mm long, soon glabrous. *Leaves*: petioles 4–8 by 5 mm, sparsely covered with appressed hairs similar to hairs on twigs to glabrous; lamina narrowly elliptic to narrowly elliptic-oblong, 10–17 by 1–2 cm (leaf index 5–8.5), membranous, greenish brown above, somewhat lighter so below, glabrous above except for primary vein densely to sparsely covered with more or less curved hairs, sparsely covered with appressed hairs to mostly glabrous and the primary vein rather densely so to glabrous below, base acute to attenuate, apex long-acute to long-acuminate (acumen 10–30 mm long or not distinct), primary vein impressed above, secondary veins distinct to rather indistinct, 15–17 on either side, often loop-forming, shortest distance between loops and margin 1–2 mm, tertiary venation reticulate, domatia absent. *Flowers* solitary, among leaves, supra-axillary; pedicels ca. 30 by 1 mm, to 50 by 2 mm in fruit, sparsely covered with appressed hairs, soon glabrous; bracts minute, all basal; sepals free, broadly ovate, to ca. 2 mm long, appressed; corolla tube ca. 5 mm high, ca. 7 mm in diam., wings slightly recurved, ca. 10 by 3 mm, ≥ 2 mm thick, free part of inner petals sagittate-triangular, ca. 2 mm long and wide. *Fruit* green to yellow, globose or irregularly so, ca. 2.5 cm diam., glabrous, carpels 20–25, areoles cushion-shaped, slightly protruding. *Seeds* 6–7 by 6 mm, brown.

**Figure 4.**
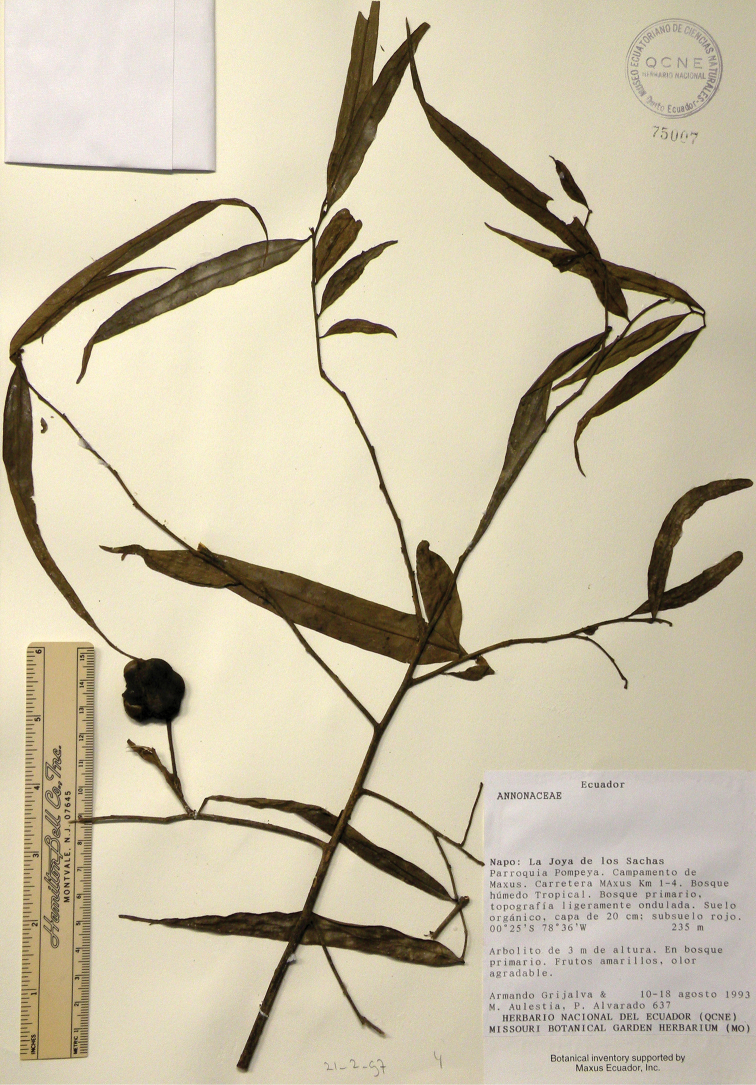
*Annonaoleifolia* Westra & H.Rainer. Fruiting specimen (*Grijalva et al. 637*, holotype QCNE).

**Figure 5.**
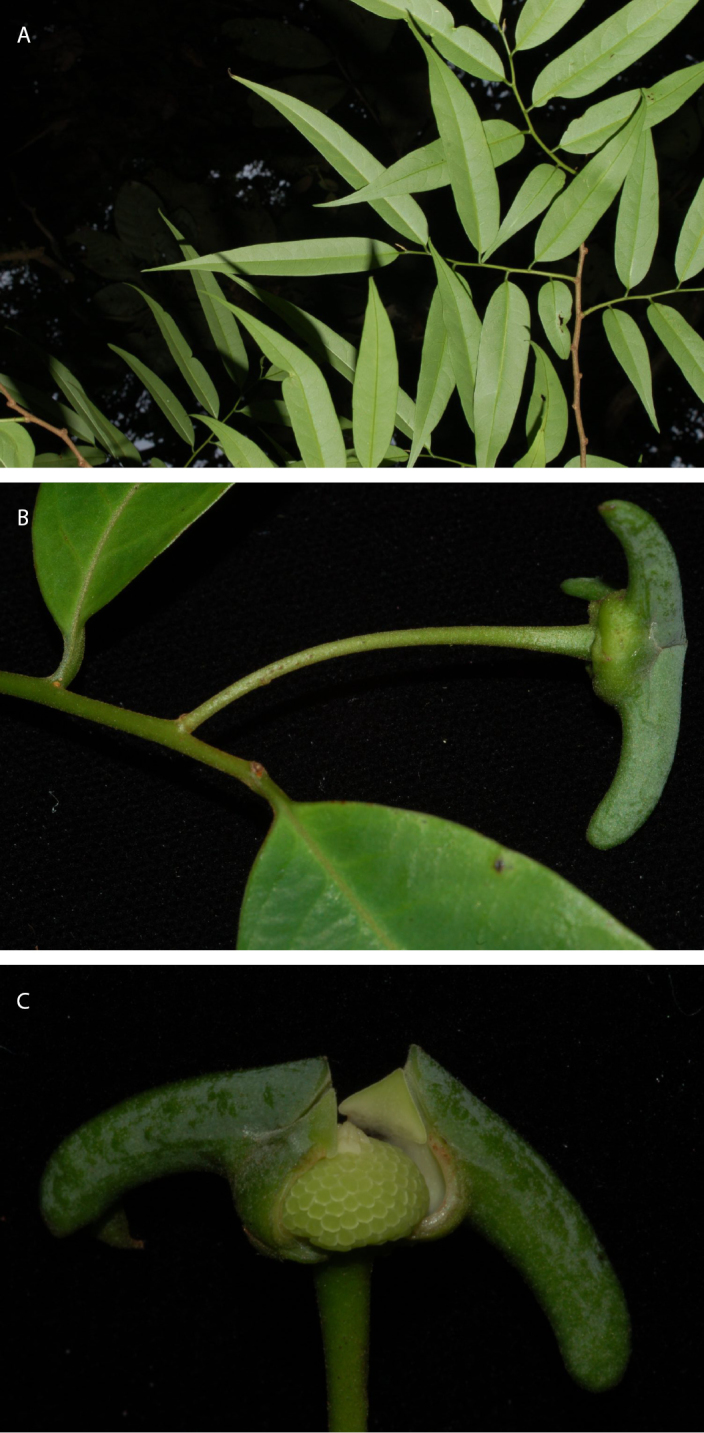
*Annonaoleifolia* Westra & H.Rainer. **A** Leaves **B** flowering branch **C** flower with part of the corolla removed to show the interior. Photographs by H. Rainer.

#### Distribution.

Ecuador (Napo), Peru (Loreto), Bolivia (Beni) (Fig. 3).

#### Habitat and ecology.

In forest. At elevations of 100–300 m; flowering: May, June; fruiting: August and September.

#### Notes.

The flower had to be described from photographs. *Annonaoleifolia* falls within the former concept of *Rollinia* because of the characteristic shape of the corolla, with the outer petals wing-like, and the whole flower suggesting a miniature propellor (see Maas et al. 1992).

Using the key to the species in the monograph of *Rollinia* (Maas et al. 1992), *Annonaoleifolia* ends near *Rollinasericea* = *Annonaneosericea*, but it is immediately distinct from that species by the very low number of carpels (≤25 vs. ≥100, respectively).

#### Etymology.

‘Oleifolia’ = with leaves resembling those of *Oleaeuropaea* L., the Olive Tree.

#### Preliminary IUCN conservation status.

EN B2ab(iii). The EOO (126.356 km2) was too large to classify as threatened, but AOO (24 km2) would classify as Endangered. It was determined that this species occurs in 5 locations. Although the species occurs within national parks in Ecuador, it is also found in heavily fragmented forest regions. Since the current population size and population trend of this species are unknown, we have classified it as Endangered.

#### Other specimens examined.

**ECUADOR. Napo**: La Joya de los Sachas, Cantón Pompeya, 00°25'S, 78°37'W, 14 Sep 1992, *Gudiño & Grefa 1775* (MO, QCNE). **PERU**. **Loreto**: Prov. Maynas, Distr. Sargento Lores, Constancia Norte, 04°07'S, 72°55'W, 11 Apr 1997, *Vásquez et al. 22963* (MO). **BOLIVIA**. **Beni**: Prov. Vaca Diez, Cachuela Esperanza, along Río Beni, 10°32'S, 65°36'W, 9 Nov 2001, *Chatrou et al. 417* (U).

### Guatteria aliciae

Taxon classificationPlantaeMagnolialesAnnonaceae

Maas & Erkens sp. nov.

urn:lsid:ipni.org:names:77199052-1

Figs 6 , 7 , 8

#### Diagnosis.

*Guatteriaaliciae* is similar to *Guatteriatenera* R.E.Fr. in terms of its very small and narrow leaves that are not verruculose, straight young twigs, and secondary veins that are impressed to raised on the upper side of the lamina, but it is distinct from that species by long-pedicellate flowers (20–45 vs. 10–20 mm long) and longer petioles (5–10 vs. 2–5 mm long) and almost smooth (to slightly pitted) seeds.

#### Type.

PANAMA, Veraguas: Parque Nacional Santa Fé, La Sabaneta, E0501556 N0959877, 1000 m, 16 Jul 2009, *Ibañez et al. 5799* (holotype: MO! [MO6619251]; isotype: L!).

#### Description.

*Tree* 4–6 m tall; young twigs sparsely covered with appressed hairs, soon glabrous. *Leaves*: petioles 5–10 by 2 mm; lamina narrowly elliptic to narrowly oblong-elliptic, 8–12 by 3–4 cm (leaf index 2.6–3.6), chartaceous, not verruculose, dull, greyish above, brown below, glabrous above, sparsely to densely (large veins) covered with appressed hairs below, base acute to obtuse, or attenuate, apex acuminate (acumen 5–15 mm long), primary vein impressed above, secondary veins distinct, 10–12 on either side of primary vein, slightly raised above, smallest distance between loops and margin ca. 2 mm, tertiary veins indistinct, flat above, reticulate. *Flowers* solitary or rarely in 2-flowered inflorescences in axils of leaves or on leafless branchlets; flowering and fruiting pedicels 20–45 by 1 mm, sparsely to rather densely covered with appressed hairs, articulated at 0.2–0.3 from the base; bracts (4-)6–7, soon falling, the basal ones (one seen) broadly ovate, ca. 1 mm long, the 2 upper ones not seen; flower buds depressed ovoid; sepals free, broadly ovate-triangular, 5–6 by 5–6 mm, appressed, outer side rather densely covered with appressed hairs; petals green to yellowish green *in vivo*, oblong-elliptic, 10–15 by 4–6 mm, outer side densely covered with appressed hairs; stamens ca. 2 mm long, connective shield densely papillate. *Monocarps* ca. 20, green *in vivo*, black *in sicco*, ellipsoid, 9–10 by 4 mm, glabrous, apex apiculate (apiculum <0.5 mm long), wall ca. 0.2 mm thick, stipes red *in vivo*, 10–15 by 1 mm. *Seed* ellipsoid, ca. 10 by 4 mm, brown, surface smooth to slightly pitted, raphe raised.

**Figure 6.**
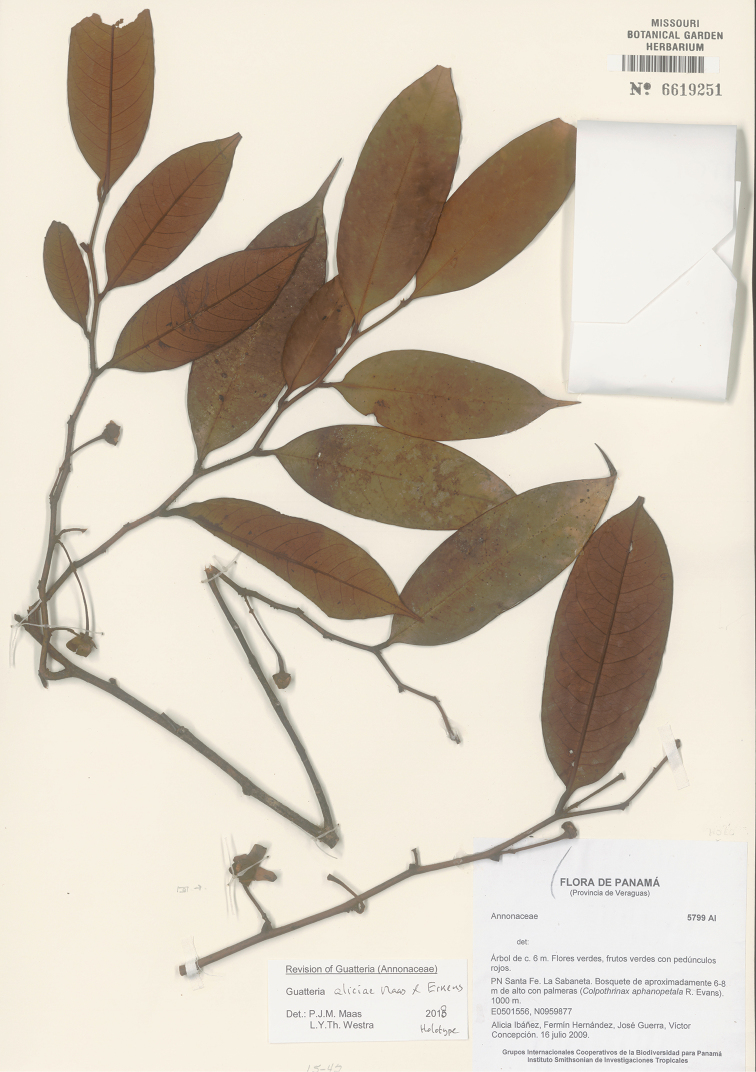
*Guatteriaaliciae* Maas & Erkens. Flowering branch (*Ibañez et al. 5799*, holotype MO).

**Figure 7.**
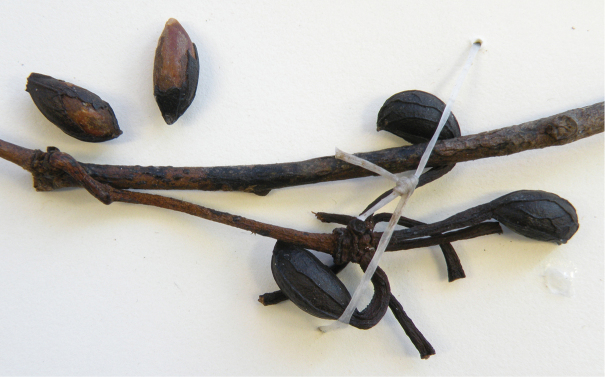
*Guatteriaaliciae* Maas & Erkens. Fruiting branch, detail (*Ibañez et al. 5813*, MO).

**Figure 8.**
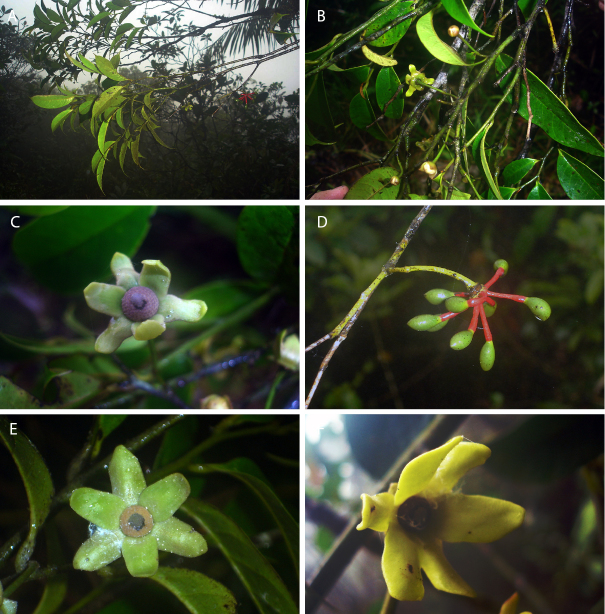
*Guatteriaaliciae* Maas & Erkens. **A, B** Flowering branch **C** flower seen from above **D** young fruit **E** flower seen from aside **F** flower seen from above (**A–D***Ibañez et al. 5799***E***Ibañez et al. 5770***F***Ibañez et al. 5813*).

#### Distribution.

**Figure 9.**
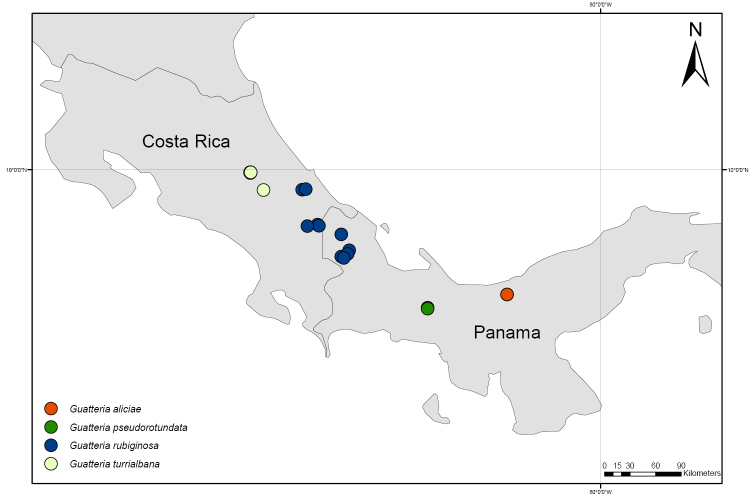
Distribution map of *Guatteriaaliciae*, *G.pseudorotundata*, *G.rubiginosa*, and *G.turrialbana*.

Panama (Veraguas) (Fig. 9).

#### Habitat and ecology.

In low forest of 6–8 m tall, with the palm species *Colpothrinaxaphanopetala* R.Evans. At an elevation of ca. 1000 m. Flowering and fruiting: July.

#### Notes.

*Guatteriaaliciae* is named in honour of its collector Alicia Ibañez, who assisted us in all kinds of ways in 2006, during our visit to Panama. This species is only known from one locality in the Panamanian province of Veraguas.

#### Preliminary IUCN conservation status.

DD. This species is only known from one locality with three collections. Therefore AOO and EOO could not be calculated. Given that the species occurs in Santa Fé National Park we assume that currently there are no major threats. However, since the current population size and population trend of this species are unknown, it was assessed as Data Deficient.

#### Other specimens examined.

**PANAMA**. **Veraguas**: Parque Nacional Santa Fé, La Sabaneta, E0501556 N0959877, 1000 m, 16 Jul 2009, *Ibañez et al. 5770* (MO), *5813* (MO, 2 sheets).

### Guatteria attenuata

Taxon classificationPlantaeMagnolialesAnnonaceae

Maas & Westra sp. nov.

urn:lsid:ipni.org:names:77199053-1

Fig. 10

#### Diagnosis.

Resembling the Amazonian species *G.modesta* Diels by the long-attenuate leaf base, but differing by a shorter petiole (2–5 vs. 5–10 mm long), and distinct secondary and tertiary veins which are strongly raised above vs. inconspicuous and flat to slightly raised above.

#### Type.

BRAZIL, Bahia: Almadina, Serra do Sete-Paus, Rodovia de Almadina para Ibitupã, entrada à esquerda ca. 5 km Fazenda Cruzeiro do Sul, ca. 8 km da entrada do ramal, 14°44'06"S, 39°41'46"W, 300 m, 3 Mar 2005, *Fiaschi et al. 2735* (holotype: NY! [NY01196019]; isotypes: RB! [RB427393], U! [U0248902]).

#### Description.

*Tree* ca. 35 m tall, to ca. 58 cm diam.; young twigs densely covered with half-appressed hairs. *Leaves*: petioles 2–5 by 0.5–1 mm; lamina narrowly elliptic, 6–12 by 2–3 cm (leaf index 3.5–4), chartaceous to coriaceous, discolorous, greyish green above *in sicco*, brown below *in sicco*, sparsely covered with appressed hairs above, mainly along primary vein, rather densely covered with appressed hairs below, base long-attenuate, basal margins revolute, apex very shortly and bluntly acuminate (acumen 1–3 mm long), primary vein impressed above, secondary veins 13–18 on either side of primary vein, strongly raised above, smallest distance between secondary veins and margin 2–3 mm, tertiary veins strongly raised on both sides, strongly reticulate. *Inflorescence* axillary, 1–2-flowered; pedicels 8–20 by 1–2 mm, densely covered with half-appressed, white hairs, articulated at 0.3–0.4 from the base; bracts 5–7, depressed ovate, 1–2 mm long, outer side densely covered with half-appressed, white hairs; flower buds not seen; sepals free, deltate, 4–5 by 4–5 mm, reflexed, inner and outer side densely covered with appressed and erect, curly, greyish hairs; petals greenish, maturing yellowish cream *in vivo*, narrowly elliptic to obovate, 10–16 by 6–8 mm, inner and outer side densely covered with appressed and erect, curly, greyish hairs; stamens 1–1.5 mm long, connective shield discoid, glabrous. *Monocarps* and *seeds* not seen.

**Figure 10.**
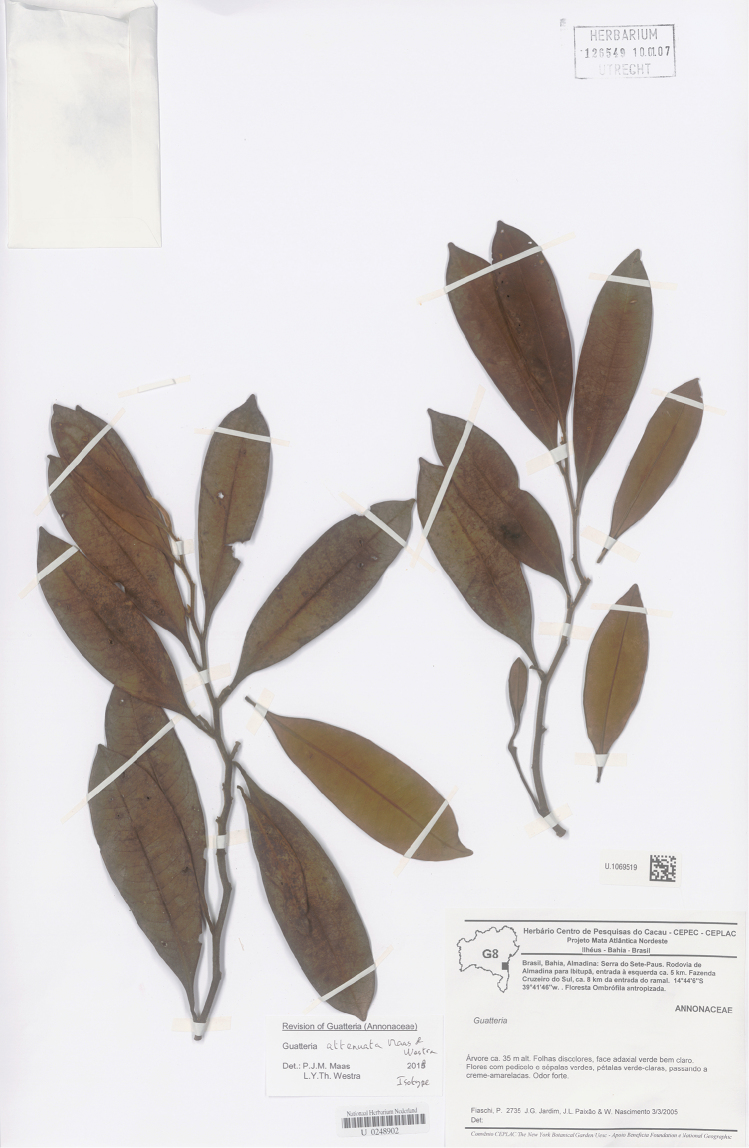
*Guatteriaattenuata* Maas & Westra. Flowering branch (*Fiaschi et al. 2735*, isotype U).

#### Distribution.

Brazil (Bahia) (Fig. 3).

#### Habitat and ecology.

In non-inundated, Atlantic rain forest. At an elevation of ca. 300 m. Flowering: March; fruiting: not recorded.

#### Notes.

*Guatteriaattenuata*, only known from the type collection and a second sterile collection from the same tree, is according to the label a tree of 35 m high, a size rarely seen in the genus. Also the long-attenuate leaf base is a rare feature in the *Guatteria*, although in *G.modesta* from the Amazon region, the base is attenuate.

#### Preliminary IUCN conservation status.

DD. This species is only known from one locality and the current population size and population trend of this species are unknown. *Guatteriaattenuata* was collected in an area that shows habitat loss due to forest cover loss and this is therefore a possible threat for this species. Nonetheless, this species was assessed as Data Deficient given the overall lack of data of this species.

#### Other specimen examined.

**BRAZIL. Bahia**: Almadina, Serra do Sete-Paus, 6 km de Almadina, na estrada para Ibitupã, então 7 km N para a comunidade de Sete-Paus, na nascente do rio Almadina, 14°44'S, 39°42'W, 19 Jul 2005, *Lobão et al. 735* (RB).

#### Field observations.

According to the label of the type collection, the flowers emit a strong scent (“odor forte”).

### Guatteria kamakusensis

Taxon classificationPlantaeMagnolialesAnnonaceae

Maas & Westra sp. nov.

urn:lsid:ipni.org:names:77199054-1

Fig. 11

#### Diagnosis.

*Guatteriakamakusensis* resembles *G.schomburgkiana* Mart. by solitary, short-pedicellate flowers in axils of leaves but differs by the connective shield of the stamens which are papillate vs. densely hairy in *G.schomburgkiana*.

#### Type.

GUYANA, Cuyuni-Mazaruni Region, 2^nd^ and 3^rd^ escarpments of Kamakusa Mt., 5°52'55.2"N, 60°6'34.5"W, 1330 m, 8 Jun 2012, *K.J. Wurdack et al. 5874* (holotype: U!; isotypes US!).

#### Description.

*Tree* to 10 m tall; young twigs densely to rather densely covered with appressed, whitish hairs to ca. 0.5 mm long, soon glabrous. *Leaves*: petioles 10–15 by 1.5–2.5 mm; lamina elliptic, 12–17 by 4.5–7 cm (leaf index 2.5–2.7), thinly coriaceous, smooth, slightly shiny above and below *in sicco*, greyish brown above *in sicco*, pale brown below *in sicco*, sparsely covered with appressed hairs to glabrous above, rather densely (primary vein) to sparsely covered with appressed, whitish hairs below, base acute, extreme base shortly attenuate, apex acuminate (acumen 10–15 mm long), primary vein impressed above, secondary veins distinct, 10–12 on either side of primary vein, slightly raised above, loop-forming in part, shortest distance between loops and margin 2–3 mm, tertiary veins mostly reticulate, slightly raised above. *Flowers* solitary in axils of leaves; pedicels 5–7 by 1.5–2.5 mm, densely covered with appressed hairs, articulated at 0.5–0.6 from the base; bracts 4–5, soon fallling, basal ones 1–1.5 mm long, the two uppermost elliptic, ca. 5 mm long; flower buds very broadly ovoid, apiculate; sepals basally connate to free, broadly triangular-ovate, 3–5 by 5–6 mm, outer side densely covered with appressed hairs; petals [as “calyces”] green *in vivo*, elliptic, ca. 10 by 5 mm, outer side densely covered with appressed hairs; stamens 80–100, yellow *in vivo*, ca. 1.5 mm long, connective shield densely covered with papillae and with few minute erect hairs; carpels 30–40, stigmas green *in vivo*. *Monocarps* and *seeds* not seen.

**Figure 11.**
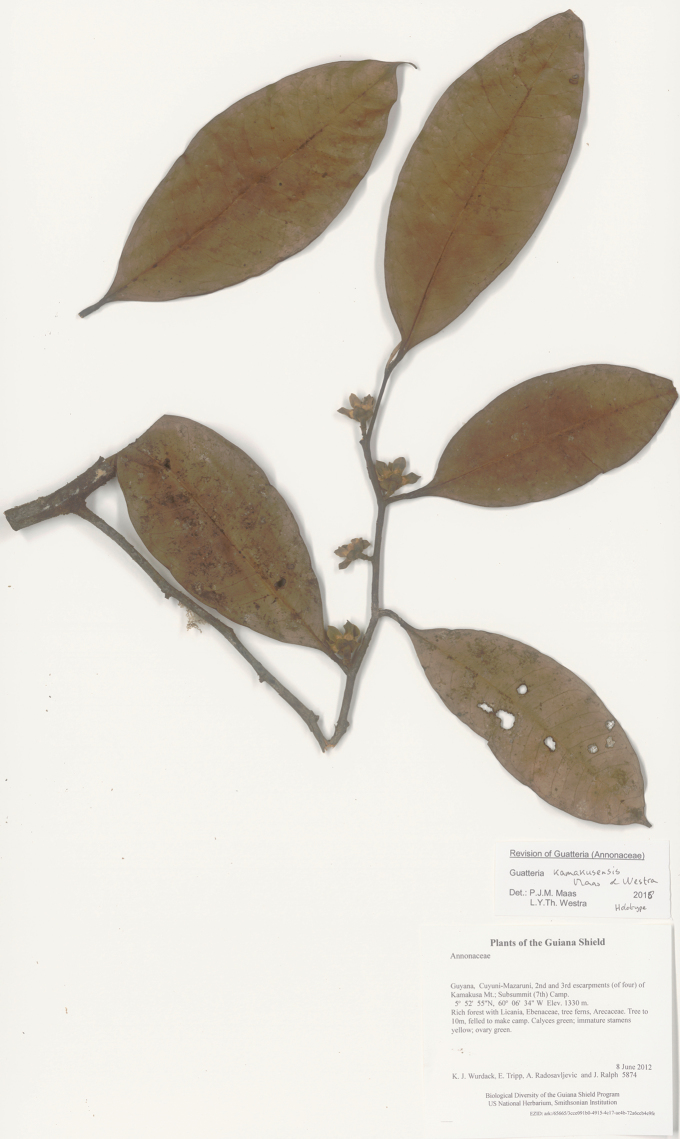
*Guatteriakamakusensis* Maas & Westra. Flowering branch (*Wurdack et al. 5874*, holotype U).

#### Distribution.

Guyana, only known from the type collection (Fig. 3).

#### Habitat and ecology.

In rich rain forest with *Licania*, Ebenaceae, tree ferns, and palms. At an elevation of ca. 1330 m. Flowering: June; fruiting: unknown.

#### Notes.

*Guatteriakamakusensis* was found at a fairly high elevation as compared to most of the *Guatteria* species in the Guianas, in a poorly collected area. It is similar to *G.schomburgkiana* Mart. in leaf shape and the short-pedicellate flowers, but the connective shield is papillate with few intermixed hairs, vs. a densely hairy connective shield in *G.schomburgkiana*. Its place remains unclear though, especially because the fruits are still lacking, and requires further research.

#### Preliminary IUCN conservation status.

DD. This species is only known from one locality in a poorly collected area and the current population size and population trend of this species are unknown. There seem to be no immediate threats to this species in terms of habitat loss. However, since no AOO and EOO could be determined or any other assessment criterium could be used, this species was assessed as Data Deficient.

### Guatteria pseudoferruginea

Taxon classificationPlantaeMagnolialesAnnonaceae

Maas & Westra sp. nov.

urn:lsid:ipni.org:names:77199055-1

Figs 12 , 13

Guatteria sp. 2 Maas & Westra, Blumea (2015) 188.

#### Diagnosis.

*Guatteriapseudoferruginea* superficially resembles the SE Brazilian *G.ferruginea* A.St.-Hil. by having young twigs densely covered with erect, brown hairs, non-verruculose leaves, and ellipsoid seeds, but it is different from that species by being not cauliflorous, and having smaller leaves (14–20 vs. 17–40 cm long), petals hairy on both sides, shorter pedicels (7–15 vs. 15–70 mm long) and seeds that are smooth vs. pitted.

#### Type.

COLOMBIA, Vichada: Gaviotas, afluente del Caño Urimica, 1 Jan 1973, *Cabrera R. 2522* (holotype: COL! [COL411248]; 2 isotypes: COL! [COL265832 and COL411249).

#### Description.

*Tree* or *shrub* 5–12 m tall, 5–15 cm diam.; young twigs densely covered with erect, brown hairs, soon glabrous. *Leaves*: petioles 4–10 by 1–2 mm; lamina narrowly elliptic, 14–20 by 4–6.5 cm (leaf index 2.8–3.5), chartaceous, not verruculose, dull, greyish above, brown below, densely covered with appressed hairs to glabrous above, sparsely covered with appressed hairs to glabrous below, base acute to slightly attenuate, apex acuminate (acumen 5–15 mm long), primary vein impressed above, secondary veins distinct, 15–17 on either side of primary vein, slightly impressed above, smallest distance between loops and margin ca. 2 mm, tertiary veins flat above, reticulate. *Inflorescence* axillary, 1–2-flowered; pedicels 7–15 by 1 mm, to 15–20 by 1–2 mm in fruit, densely covered with appressed and erect, brown hairs, articulated at ca. 0.3 from the base; bracts not seen; flower buds subglobose; sepals free, broadly ovate-triangular, 7–8 by 6 mm, reflexed, outer and inner side densely covered with appressed and erect, brown hairs, inner base glabrous; petals green *in vivo*, ovate-elliptic, 7–12 by 5–11 mm, both sides densely covered with appressed and erect, brown hairs, except for the inner glabrous base; stamens ca. 1.5 mm long, connective shield papillate. *Monocarps* 10–50, colour *in vivo* not recorded, black *in sicco*, ellipsoid to narrowly ellipsoid, 10–20 by 5 mm, glabrous, apex apiculate (apiculum < 0.5 mm long), wall ca. 0.2 mm thick, stipes 15–20 by 5 mm. *Seed* ellipsoid, dark, shiny brown, ca. 8 by 5 mm, smooth.

**Figure 12.**
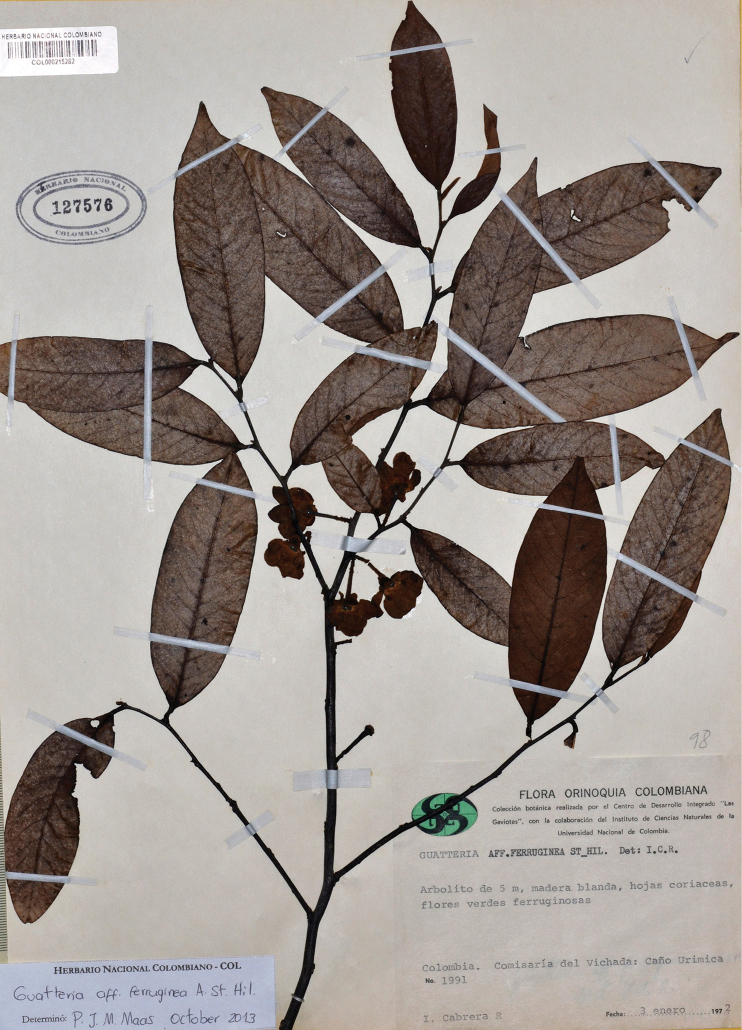
*Guatteriapseudoferruginea* Maas & Westra. Flowering branch (*Cabrera R. 1991*, COL).

**Figure 13.**
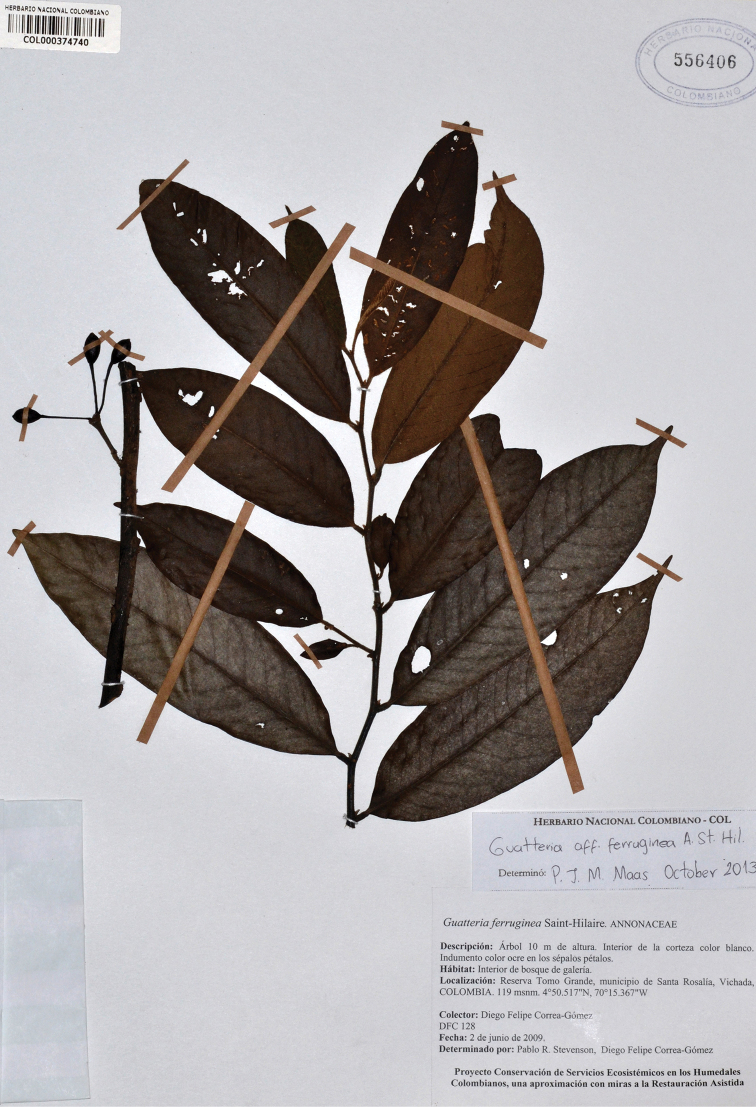
*Guatteriapseudoferruginea* Maas & Westra. Fruiting branch (*Correa-Gómez 128*, COL).

#### Distribution.

Colombia (Arauca, Meta, Vichada) (Fig. 3).

#### Habitat and ecology.

In non-inundated forest or gallery forest. At elevations of 100–1000 m. Flowering: January to April; fruiting: March, June.

#### Notes.

Specimens of *Guatteriapseudoferruginea* have previously been identified as *G.ferruginea* A.St.-Hil. from E and SE Brazil, which differs from *G.pseudoferruginea* in being cauliflorous. Both species are characterised by a dense indument of brownish, erect hairs on the twigs. However the new species differs from *G.ferruginea* by characters as given in the diagnosis, notably by petals covered with brown hairs on both sides (vs. on the outer side only in *G.ferruginea*), and by the absence of cauliflory .The description of *G.pseudoferruginea*, formerly named *Guatteria sp. 2* in the monograph of *Guatteria* (Maas et al. 2015), could be completed by the first author during a recent visit to Colombia and the COL Herbarium in Bogotá.

#### Preliminary IUCN conservation status.

EN B2ab(iii). EOO (378.742 km^2^) was too large to classify as threatened, but AOO (20 km^2^) would classify as Endangered. It was determined that this species has 5 locations almost all in heavily deforested areas outside national parks. Furthermore, because no information is available on the current population size and population trend of this species, we have classified it as Endangered.

#### Other specimens examined.

**COLOMBIA. Arauca**: Mun. Tame, Vereda Caribabare, 6°16'36.5"S, 71°46'01.4"W, 290 m, 30 Mar 2015, *Trujillo-C. & Gantiva 3298* (COL). **Meta**: San Martín, Vereda La Castañeda, Finca Santa Rosa, 3°36'51"N, 73°38'33"W, 363 m, 27 Feb 2005, *Aldana & Stevenson 10* (ANDES), *22* (ANDES, COL); forested slopes of Río Negro, ca. 20 km W of Villavicencio, along road between main highway and finca of Helmuth Schmidt, 1050 m, 23 Feb 1972, *Barclay et al. 3198* (US). **Vichada**: Caño Urimica, 3 Jan 1972, *Cabrera R. 1991* (COL); Mun. Puerto Carreño, Mata de monte grande, 5°17'00"N, 67°57'50"W, 4 Aug 1995, *Córdoba et al. 1369* (COL); Mun. Santa Rosalia, Reserva Tomo Grande, 4°50.400'N, 70°16.388'W, 124 m, 27 Apr 2009, *Correa-Gómez 87* (COL); Mun. Santa Rosalia, Reserva Tomo Grande, 4°50'517"N, 70°15'367"W, 119 m, 2 Jun 2009, *Correa-Gómez 128* (COL).

### Guatteria pseudorotundata

Taxon classificationPlantaeMagnolialesAnnonaceae

Maas & Erkens sp. nov.

urn:lsid:ipni.org:names:77199056-1

Figs 14 , 15 , 16

#### Diagnosis.

*Guatteriapseudorotundata* resembles the Panamanian *G.rotundata* Maas & Setten by its coriaceous leaves, the number of distinct secondary veins below (8–12 vs. 7–12) and the broadly ovate-triangular sepals, but it differs by the young twigs that are glabrous vs. sparsely covered with appressed hairs, slightly smaller leaves (5–9 vs. 5–14 cm long) with a mostly acute leaf apex vs. obtuse or rounded apex, the lack of any verruculae in the lamina, and longer pedicels (15–20 vs. 4–15 mm long).

#### Type.

PANAMA, Comarca Ngabe-Buglé: Nole Duima, Alto Ratón, E409440, N944626, 1590 m, 28 Nov 2011, *Pineda & Castillo 15* (holotype: MO! [MO6613500]).

#### Description.

*Tree* 6–10 m tall; young twigs glabrous. *Leaves*: petioles 2–4 by 1 mm; lamina narrowly elliptic, 5–9 by 2–3 cm (leaf index 2.5–3), chartaceous *in sicco*, coriaceous *in vivo*, not verruculose, shiny above *in vivo*, brown above, paler brown below, glabrous on both sides, base attenuate, apex obtuse or more or less acute with an obtuse extreme apex, primary vein slightly raised above, secondary veins distinct, 8–12 on either side of primary vein, raised above (but even more so below), smallest distance between loops and margin 1–2 mm, tertiary veins raised above, reticulate. *Flowers* solitary in axils of leaves; pedicels 15–20 by 0.5–1 mm to 1.5 mm diam. in fruit, rather densely to sparsely covered with appressed hairs, articulated at 0.1–0.2 from the base; bracts 5–7, soon falling, one of the lower bracts sometimes leafy, ca. 15 by 5 mm; flower buds ovoid, slightly pointed; sepals free, broadly ovate-triangular, ca. 4 by 3 mm, appressed, outer side rather densely covered with appressed, greyish white hairs, particularly towards the apex; petals greenish yellow *in vivo*, ovate-elliptic, 6–7 by 5 mm, outer and inner, side densely covered with appressed and curly, greyish white hairs, base of inner petals glabrous; stamens 1–2 mm long, connective shield papillate. *Monocarps* 10–25, green *in vivo*, black *in sicco*, narrowly ellipsoid, 13–16{-18} by 4{-6} mm, glabrous, apex apiculate (apiculum <0.1 mm long), wall 0.1–0.2 mm thick, stipes 1–4 by 1–2 mm. *Seed* narrowly ellipsoid, 13–16 by 4–5 mm, brown, rugulose.

**Figure 14.**
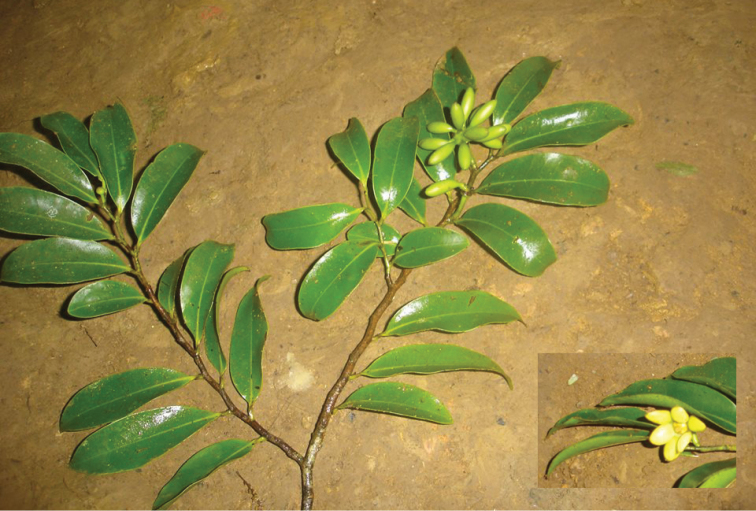
*Guatteriapseudorotundata* Maas & Erkens. Fruiting branch and flower (Maas et al. (2015): 148, Plate 7a [as *Guatteriarotundata*]).

**Figure 15.**
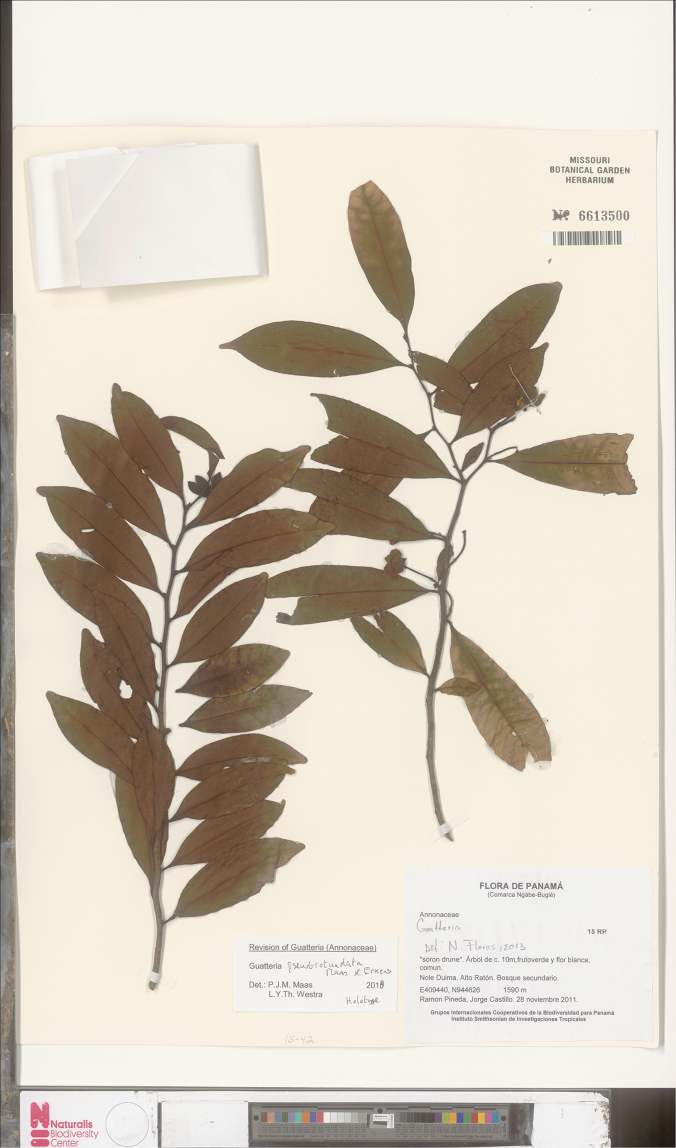
*Guatteriapseudorotundata* Maas & Erkens. Flowering branch (*Pineda et al. 15*, holotype MO).

**Figure 16.**
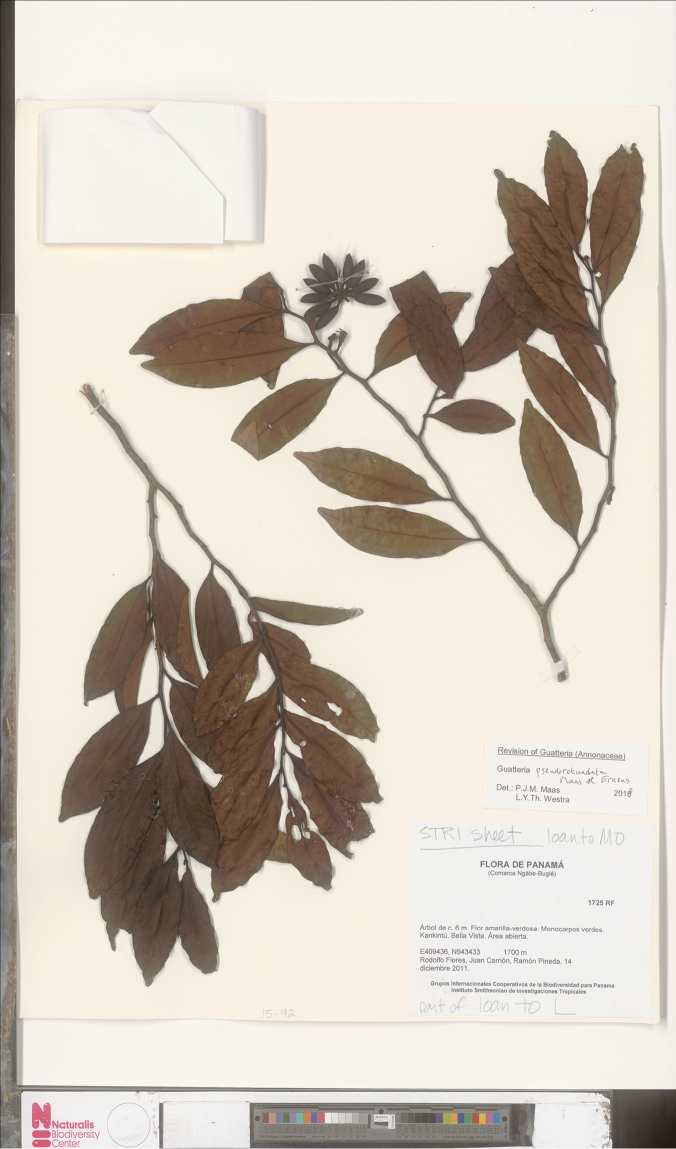
*Guatteriapseudorotundata* Maas & Erkens. Fruiting branch (*Flores et al. 1725*, STRI).

#### Distribution.

Panama (Comarca Ngabe-Buglé) (Fig. 9).

#### Habitat and ecology.

In secondary forest. At elevations of 1590–1700 m. Flowering and fruiting: November and December.

#### Vernacular names.

Panama: Soron drune.

#### Notes.

When working on the revision of *Guatteria* (Maas et al. 2015) we received photographs of flowering and fruiting specimens of a plant from Panama which seemed to match well *Guatteriarotundata* Maas & Setten, and we identified them as such and included the appropriate illustration as Pl. 7a in our work. *G.rotundata*, it should be pointed out, is unique among Central American species of *Guatteria* on account of its leaves having a rounded apex. Recently we received the corresponding herbarium material and it became clear that the photographed plant did not represent *G.rotundata* at all, but an undescribed species instead. *G.pseudorotundata* differs from *G.rotundata* by characters as given in the diagnosis, but notably the lack of verruculae in the lamina. Although more or less hidden from view in the photograph just mentioned, the leaf apex in *G.pseudorotundata* tends to be acute rather than obtuse or rounded (excl. the extreme tip) as in *G.rotundata*.

#### Preliminary IUCN conservation status.

DD. This species is only known from three nearby collections and therefore no AOO and EOO was calculated (that would constitute one location) in a region that is partially deforested. More continuous forest is, however, available nearby but it is unclear whether this species occurs there. Habitat loss because of forest cover loss is therefore a possible threat for *Guatteriapseudorotundata*. Unfortunately, no other assessment criterium could be used for this species since no information is available on the current population size and population trend of this species. Hence, this species was assessed as Data Deficient.

#### Other specimens examined.

**PANAMA**. **Comarca Ngabe-Buglé**: Kankintú, E409436, N943433, 1700 m, Dec 2011, *Carrión et al. 517* (MO), ibidem, *Flores et al. 1725* (STRI).

### Guatteria rotundata

Taxon classificationPlantaeMagnolialesAnnonaceae

Maas & Setten, Proc. Kon. Ned. Akad. Wetensch. C 91(3): 255. f. 11. 1988.

#### Notes.

In the recent revision of *Guatteria* (Maas et al. 2015) the measurements of fruits and seeds were erroneously included under *G.rotundata*. The fruiting material appeared to belong to the now described *G.pseudorotundata* and the fruits and monocarps of *G.rotundata* are still unknown.

### Guatteria rubiginosa

Taxon classificationPlantaeMagnolialesAnnonaceae

N.Zamora & Maas sp. nov.

urn:lsid:ipni.org:names:77199057-1

Fig. 17

#### Diagnosis.

*Guatteriarubiginosa* is strikingly similar to *Guatteriatalamancana* N.Zamora & Maas in terms of the presence of long-persistent, erect, brownish red to brownish hairs of 2–3 mm long on its young twigs and lower side of the lamina, but differs by the smaller petals (11–17 by 7–8 mm vs. 15–25 by 10–15 mm), smaller sepals (7–11 by 7–10 mm vs. 15–20 by 10–15 mm), and smaller monocarps (7–13 by 4–7 mm vs. 20–30 by 18–20 mm).

#### Type.

PANAMA, Bocas del Toro: Changuinola, Parque Internacional La Amistad (PILA), Rancho Santín, 9°06'41.9"N, 82°40'03.7"W, 1340 m, 31 Jul 2008, *Monro et al*. *6108* (holotype: CR!; isotypes: BM, MO! [MO2494703]).

#### Description.

*Tree* 7–20 m tall; young twigs somewhat zigzagging, densely covered with long-persistent, erect, brownish red to brown hairs to 3 mm long. *Leaves*: petioles 0–3 mm long, densely covered with erect, brownish hairs; lamina narrowly ovate to narrowly oblong-elliptic, (5-)7–21 by (2.5-)3–5.5 cm (leaf index 3.2–3.6), chartaceous, densely verruculose on both sides, dull or dark glossy green above, brown to greenish brown below, densely covered with erect, brown to reddish brown hairs on both sides, but becoming almost glabrous above, except for the primary vein, base acute to obtuse, often oblique, apex acute to acuminate (acumen 10–15 mm long), primary vein flat above, secondary veins distinct, flat to slightly raised above, 10–20 on either side of primary vein, smallest distance between loops and margin 2–3 mm, tertiary veins inconspicuous, flat above. *Flowers* solitary in axils of leaves; pedicels 5–15 by 1–2 mm, to 20 by 3 mm in fruit, articulated at ca. 0.5 from the base, densely covered with erect, brown or reddish brown hairs; bracts soon falling, not seen; flower buds depressed ovoid; sepals free, broadly ovate-triangular, 7–11 by 7–10 mm, apex acuminate, outer side densely covered with appressed, brown hairs, inner side glabrous, conspicuously verruculose; petals pale yellow or cream *in vivo*, ovate to oblong-ovate, 11–17 by 7–10 mm, outer side densely covered with appressed hairs; stamens 1–2 mm long, connective shield glabrous. *Monocarps* 25–30, green *in vivo*, black *in sicco*, ellipsoid to narrowly ellipsoid, sometimes laterally compressed, 7–13 by 4–7 mm, glabrous, apex apiculate (apiculum <0.5 mm long), wall 0.2–0.3 mm thick, stipes 1–8 by 2–3 mm. *Seed* ellipsoid, 9–11 by 5–6 mm, dark, dark brown to reddish brown, rugose to rugulose, raphe impressed.

**Figure 17.**
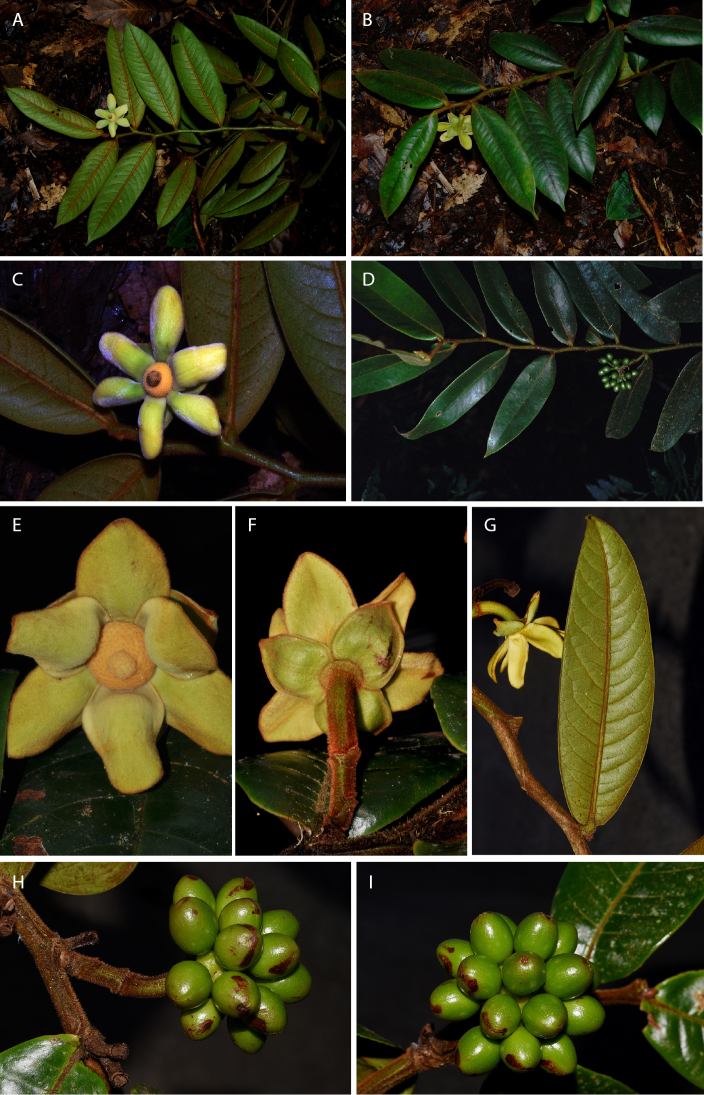
*Guatteriarubiginosa* N.Zamora & Maas. **A–C** Flowering branch **D** fruiting branch **E–G** flowers in different views **H, I** young fruit. (**A–C***Monro et al. 6108***D***Monro & Cafferty 4925***E, F***Estrada et al. 5979*; **G–I***Estrada et al. 6406*).

#### Distribution.

Costa Rica and Panama (Fig. 9).

#### Habitat and ecology.

In wet forest, sometimes cloud forest. At elevations of 400–1850 m on the Caribbean slope of the Talamanca mountain range. Flowering: March and July; fruiting: March, April, and July.

#### Notes.

*Guatteriarubiginosa* can be recognised by its young twigs and the lower side of the lamina which are densely covered with long-persistent, erect, brown to reddish brown hairs. Moreover, the leaves are subsessile and densely verruculose on both sides. The monocarps are ellipsoid and shortly stipitate. Some material of *G.rubiginosa* was confused or misidentified as *G.talamancana* N.Zamora & Maas, due to the high resemblance of the vegetative parts of both species, especially the indument. Moreover, *G.rubiginosa* differs greatly in its much wetter habitat type on the Caribbean slope of the Talamanca Costa Rica-Panama mountain range.

Some Costa Rican collections of *G.rubiginosa* were previously assigned to the Colombian *G.elegantissima* R.E.Fr. (Zamora et al. 2000), which is a species that is very similar but it is restricted to tropical rain forest of the lowland Pacific coast of Colombia (0–350 m). It has much narrower leaves (width 2–3.5 vs. 3–5.5 cm in *G.rubiginosa)* and longer pedicels (20–40 vs. 5–20 mm).

#### Preliminary IUCN conservation status.

LC. It was determined that this species has 5 locations but all of them lie within the boundaries of La Amistad International park and the National Park Palo Seco. Currently, no major threats to this species are known but also no information is available on the current population size and population trend of this species. Based on both EOO (1.583 km2) and AOO (40 km2) this species would classify as Endangered but given that we do not see any immediate threats to the size and quality of the distributional range and habitat we assess it as Least Concern.

#### Other specimens examined.

**COSTA RICA**. **Limón**: Cantón de Talamanca, Coriña, base y ladera intermedia del Cerro Cruibeta, 9°25'15"N, 82°59'00"W, 700 m, 19 Jul 1989 (fl, fr), *Herrera 3307* (CR, MO, U); Sukut, de las juntas de Río Urén y Río Sukut 1.5 km aguas arriba sobre éste, 9°24'30"N, 82°58'10"W, 400 m, 9 Jul 1989 (fl), *Herrera 3228* (CR); Bratsi, Amubri, Alto Lari, Kivut, cuenca superior del Río Dapari, 9°24'15"N, 83°05'30"W, 1200 m, 9 Mar 1992 (fl), *Herrera 5249* (CR, U); Cantón de Limón, El Progreso, siguiendo el sendero de la avioneta, por la fila entre 1000 m y los 1300 m. Fila Matama, Valle de la Estrella, 9°47'18"N, 83°08'45"W, 1150 m, 21 Apr 1989 (sterile), *Herrera & Chacón 2701* (CR, U); El Progreso, entre Cerro Muchilla y Cerro Avioneta, Fila Matama, siguiendo la fila y los flancos. Valle de la Estrella, 9°47'40"N, 83°06'30"W, 850 m, 8 Apr 1989 (flower buds), *Herrera & Madrigal 2560* (CR). **PANAMA**. **Bocas del Toro**: Campamento la pata del Cedro como a 800 m hacia arriba, 9°03.966'N, 82°43.931'W, 1525 m, 10 Mar 2004 (fr), *Alfaro & Monro 5445* (INB); Campamento de Lucho, 9°05.052'N, 82°44.733'W, 1850 m, 17 Mar 2004 (fr), *Alfaro & Monro 5577* (INB); Caribbean slopes of Cerro Fabrega at foot of Falso Fabrega, in Palo Seco Reserve, second northernmost tributary of Culubre river, Pavón Camp, 9°09.51'N, 82°39.41'W, 1300 m, 23 Mar 2005 (fr), *Monro & Cafferty 4925* (CR); Changuinola Parque Internacional La Amistad (PILA), 10 km del refugio de guardaparques de Uri, 9°04'09"N, 82°42'28"W, 15 Apr 2008 (fr), *De Serdas et al. 588* (CR). **Chiriquí**: Gualaca, Fortuna Forest Reserve of La Fortuna Watershed, close to Fortuna Dam, forest plot of Jim Dalling, 8°40'N, 82°13'W, 1150 m, 25 May 2004 (sterile), *Maas et al. 9516* (INB, U).

### Guatteria turrialbana

Taxon classificationPlantaeMagnolialesAnnonaceae

N.Zamora & Erkens sp. nov.

urn:lsid:ipni.org:names:77199058-1

Fig. 18

#### Diagnosis.

*Guatteriaturrialbana* resembles *Guatterialucens* Standl. by the narrowly elliptic to narrowly obovate leaves but differs markedly by coriaceous vs. chartaceous leaves, the primary vein of which is flat to slightly raised (vs. impressed) above, and having yellow to creamy yellow, broadly ovate to orbiculate petals (vs. orange or yellowish orange and mostly oblong to obovate petals).

#### Type.

COSTA RICA, Cartago: Turrialba, Área de Conservación Codillera Volcánica Central, Monumento Nacional Guayabo, Sendero natural, 1133 m, 9°58'15.2"N, 83°41'17.9"W, 7 Jul 2018 (fl), *Zamora & Espinoza 10363* (holotype: CR!; isotypes: B, L!, MO!).

#### Description.

*Tree* 5–20 m tall, 20–50 cm diam.; young twigs slightly zig-zagging, sparsely covered with appressed hairs, soon glabrous. *Leaves*: petioles 5–10 by 1 mm; lamina narrowly elliptic to narrowly obovate, (10-)13–23 by 4–8.5 cm (leaf index 2.8–4.2), coriaceous, not verruculose, shiny above, grey to greyish green above, grey to greyish brown below, glabrous above, sparsely covered with appressed hairs below, mainly along primary vein, base long-acute to attenuate, apex acuminate (acumen 5–10 mm long) to acute, primary vein flat to slightly raised above, secondary veins distinct, 10–15 on either side of primary vein, slightly raised above, smallest distance between loops and margin 1–3 mm, tertiary veins strongly raised above, reticulate. *Inflorescence* 1–2-flowered, in axils of leaves or on leafless branchlets; pedicels 10–30(-40) by 0.5–1 mm, 1.5–3 mm diam. in fruit, rather densely covered with appressed hairs to glabrous, articulated at 0.4–0.5 from the base; bracts 5–6, soon falling, not seen; flower buds ovoid, slightly pointed; sepals free, broadly ovate-triangular to ovate-triangular, 5–8 by 3–8 mm, reflexed, outer side densely covered with appressed hairs; petals green, maturing yellow to cream *in vivo*, broadly ovate to orbicular, 10–15{-24} by 9–10{-18} mm, outer and inner side densely covered with appressed, greyish hairs; stamens ca. 2 mm long, connective shield papillate. *Monocarps* 30–100, green *in vivo*, black *in sicco*, ellipsoid, 8–11 by 3–6 mm, glabrous, apex rostrate to apiculate (apiculum ca. 1 mm long), wall 0.1–0.2 mm thick, stipes 5–10 by 1 mm. *Seed* ellipsoid, 7–9 by 3–5 mm, brown, pitted, raphe not distinct from rest of seed.

**Figure 18.**
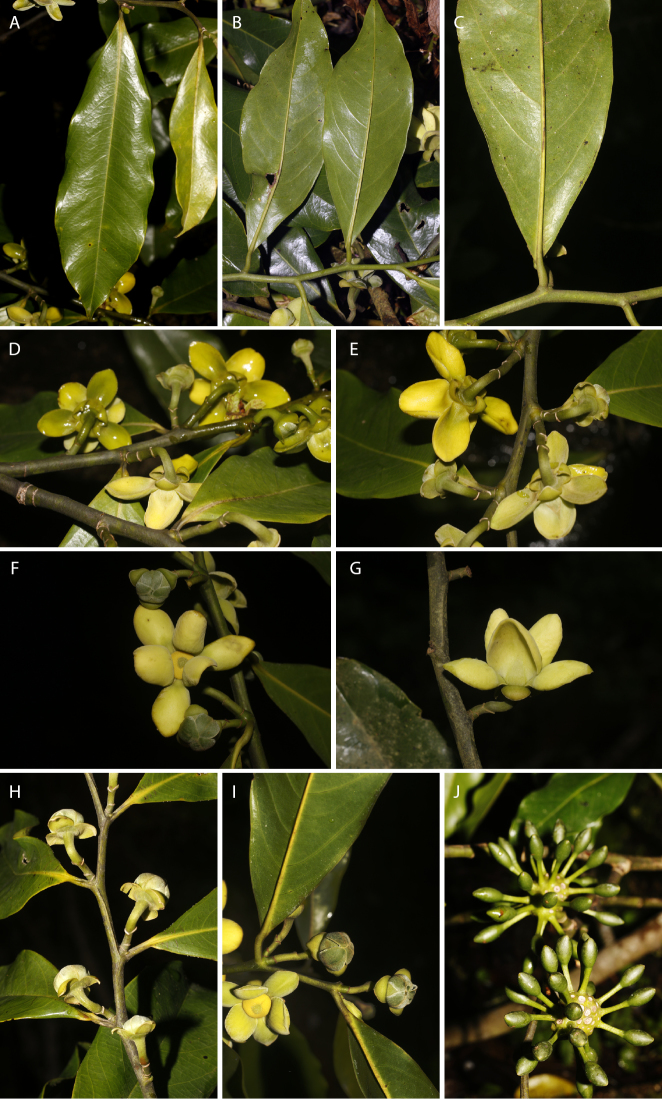
*Guatteriaturrialbana* N.Zamora & Erkens. **A** Upper side of leaf **B** lower side of leaf **C** leaf base **D, E** flowering branches **F** flower seen from above **G** flower seen from aside **H, I** flower buds **J** young fruit. (**A–J***Zamora & Espinoza 10357* and *10363*).

#### Distribution.

Costa Rica, Caribbean slope (Fig. 9).

#### Habitat and ecology.

In wet forest. At elevations of (700-)1000–1200(-1800) m, Flowering: January, May, and July; fruiting: March, October, November and December.

#### Notes.

*Guatteriaturrialbana* can be distinguished by its essentially glabrous, dark green, shiny and conspicuously reticulate leaves on both sides, especially upon drying, lamina commonly narrowly obovate with a flat to slightly raised primary vein above. The rostrate to apiculate apex of the monocarps is also a characteristic feature. Material of this species has previously been identified as *G.lucens* Standl. However, the lamina is not verruculose in *G.turrialbana* vs. up to densely verruculose in *G.lucens*. Other differences can be found in the monocarps: *G.turrialbana* has rostrate to apiculate ones vs. ellipsoid ones in *G.lucens*. Lastly, *G.lucens* is a lowland (0–900 m) species whereas *G.turrialbana* has mainly been recorded at higher elevations (Maas et al. 2015).

#### Preliminary IUCN conservation status.

EN B1ab(iii)+2ab(iii). Both EOO (10 km2) and AOO (8 km2) would classify as Endangered. It was determined that this species has 3 locations,all of them in heavily deforested areas. Deforestation is therefore a major threat to this species and habitat quality is expected to decline in the near future. No information is available on the current population size and population trend of this species. Given all this we assessed this species as Endangered.

#### Other specimens examined.

**COSTA RICA. Cartago**: Turrialba, Monumento Nacional Guayabo, Santa Teresita, sobre ríos Guayabo, Lajas y Torito, 9°57'50"N, 83°41'30"W, 700–1800 m, 8 May 1992 (fl), *Rivera 1693* (CR); Turrialba, Monumento Nacional Guayabo, 9°58'20"N, 83°41'45"W, 1100 m, 9 Oct 1993 (fr), *Vargas et al. 1492* (CR); Turrialba, Monumento Nacional Guayabo, cuenca del Río Reventazón, 9°58'19.7"N, 83°41'31.9"W, 1100–1200 m, 15 Mar 2003 (fr), *Kriebel 2977* (CR, L); Turrialba, Área de Conservación Codillera Volcánica Central, Guayabo, Guayabito de Santa Cruz, a lo largo del camino principal a Guayabo, 9°58'59"N, 83°42'54"W, 1350 m, 7 Jul 2018 (fl), *Zamora & Espinoza 10357* (CR); Turrialba, Jicotea, Finca de Israel Martínez, 9°47'05"N, 83°33'15"W, 1100–1200 m, 7 Dec 1994 (fr), *Cascante et al. 432* (CR). **Limón**: entre Dabagri y Sacabico y los bordes del mismo, 7 Nov 1984 (fr), *Gómez et al. 23305* (CR, U).

### Klarobelia rocioae

Taxon classificationPlantaeMagnolialesAnnonaceae

Chatrou sp. nov.

urn:lsid:ipni.org:names:77199059-1

Figs 19 , 20

#### Diagnosis.

*Klarobeliarocioae* is distinct from congeneric species by the combination of comparatively large leaves and large monocarps, and flowers that are hairy on the outer side.

#### Type.

PERU, Pasco: Prov. Oxapampa, Distr. Palcazú, Comunidad Nativa Alto Lagarto, 10°06'15"S, 75°33'01"W, 800 m, 2 Jul 2007, *Rojas & Ortíz 4243* (holotype: HOXA!; isotypes: MO! [MO2465956], USM, WAG!).

#### Description.

*Tree* 2–5 m tall; young twigs, lower side of petioles, and lower side of primary vein glabrous, sometimes sparsely covered with pale, appressed hairs 0.1–0.2 mm long. *Leaves*: petioles 8–12 by 2–4 mm, verrucose to rugulose, distinctly black; lamina elliptic to narrowly elliptic, 17–35 by 6.5–11.5 cm (leaf index 2.6–3.7), chartaceous, greyish to brownish green above, dark olive green to brown below, glabrous on both sides, primary vein impressed (to flat) above, base cuneate to obtuse, rarely rounded, apex acuminate (acumen 5–20 mm long) to bluntly acute, secondary veins 8–9 on either side of primary vein, distance between secondary veins 25–50 mm, angles with primary vein (45-)60–80°, loop-forming at (right-)obtuse angles, distance between loops and margin 4–8 mm, tertiary veins raised above, reticulate. *Flowers* solitary, on leafy twigs, rarely on older branchlets; short shoot and bracts rather densely covered with reddish brown, appressed hairs 0.1–0.2 mm long; short shoot 3–4.5 by 1–1.5 mm, to 3 mm in diam. when fruiting; bracts up to 3 on short shoot, 1.5–2 by 1 mm wide, apex obtuse, soon falling off; pedicels 14–18 by 1(-2) mm, to ca. 28 by 4 mm in fruit, sparsely covered with reddish brown, appressed hairs 0.1–0.2 mm long; flowers bisexual or male, plant androdioecious; flower buds (sub)globose, 12–14 mm in diam.; petals yellow to yellowish-cream *in vivo*, brown to black *in sicco*; sepals free, broadly ovate, 7–8 by 6–8 mm, black *in sicco*, ciliate, outer side glabrous, inner side sparsely to rather densely covered with yellowish to reddish brown, appressed hairs 0.1–0.2 m long; petals (broadly) ovate to (broadly) elliptic, outer petals 15–17 by 12–14 mm, slightly concave, densely covered with hairs on both sides apart from basal, central part of outer side, inner petals 15–18 by 10–13 mm, densely hairy on both sides, strongly concave; stamens ca. 50 on bisexual flowers, ca. 200 on male flowers, 1.8–2.5 mm long, thecae 0.8–1.0 mm long, apical prolongation of connective papillate; carpels ca. 110, ovaries 1.8–2.2 mm long, glabrous, stigmas 0.6–0.8 mm long, densely covered with yellowish brown hairs ca. 0.2 mm long; flowering receptacle dome-shaped, ca. 4 by 5 mm, glabrous. *Monocarps* up to 30, yellow to orange *in vivo*, dark brown *in sicco*, (oblong-)ellipsoid, slightly asymmetrical, 20–27 by 10–12 mm, glabrous, verrucose, wall 0.1–0.2 mm thick, angles between longitudinal axis of monocarps and stipes 0–90°, stipes 25–37 by 1–1.5 mm, to 1.5(-2) mm in diam. apically, fruiting receptacle irregularly subglobose to transverse ellipsoid, 5–12 by 5–13 mm. *Seed* (oblong-) ellipsoid, 20–27 by 10–12 mm, pale golden-brown, shiny, raphe slightly sinuous, ruminations lamelliform with four, thin transverse plates.

**Figure 19.**
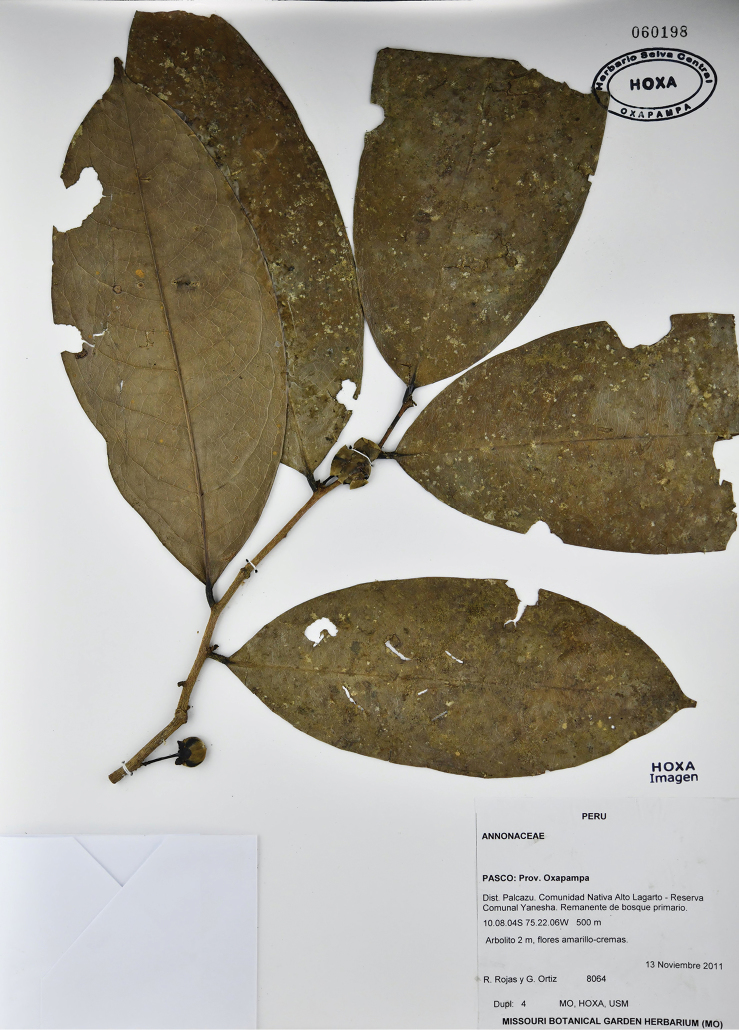
*Klarobeliarocioae* Chatrou. Flowering specimen (*Rojas & Ortiz 8064*, HOXA).

**Figure 20.**
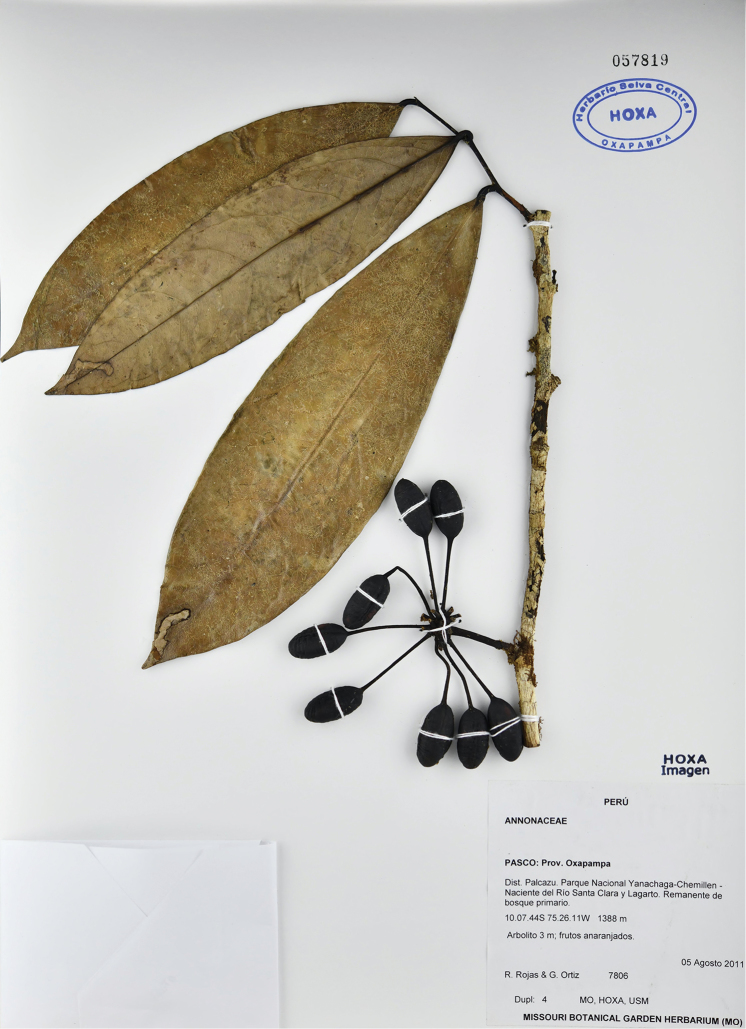
*Klarobeliarocioae* Chatrou. Fruiting specimen (*Rojas & Ortiz 7806*, HOXA).

#### Distribution.

Peru, only known from a small area in the department of Pasco, districts of Palcazú and Villa Rica (Fig. 3).

#### Habitat and ecology.

In primary forest. At elevations of ca 500–1400 m. Flowering: October and November; fruiting: between February and August (mature fruits collected in July and August).

#### Notes.

*Klarobeliarocioae* is easy to recognise through its combination of large leaves, hairy flowers, and relatively large and ellipsoid monocarps. Of the Amazonian species of *Klarobelia* Chatrou, *K.napoensis* Chatrou has comparably large leaves, but differs in the flowers that are glabrous on the outer side, and in the globose monocarps. *K.pandoensis* Chatrou and *K.pumila* Chatrou are two other Amazonian species that share a small habit with *K.rocioae* (Chatrou 1998; Chatrou and Pirie 2003). With *K.rocioae* they share outer petals that are hairy on the outer side, giving the flowers a brown appearance rather than glabrous flowers in other species of *Klarobelia* that appear black when dried. *K.pumila* Chatrou, can easily be distinguished from *K.rocioae* by the smaller leaves (12–16 by 4–6 cm vs. 17–35 by 6.5–11.5 cm in *K.rocioae*) and smaller monocarps (9–15 by 6–8 mm vs. 20–27 by 10–12 mm). *K.pandoensis* Chatrou can be distinguished from *K.rocioae* by the smaller leaves (8.5–12.5 by 3–4.5 cm) and smaller monocarps (15–18 by 6–10 mm) too. Additionally, *K.pandoensis* can easily be distinguished from the two other short stature species by the dense indument on petioles, young twigs and inflorescences axes (glabrous to sparsely hairy in *K.rocioae* and *K.pumila*). The three species have non-overlapping areas of distribution in the Amazonian lowlands and Andean foothills of central and southern Peru and northern Bolivia. Next to *K.napoensis* and *K.peruviana* (R.E.Fr.) Chatrou, *K.rocioae* is a third species within the genus reaching altitudes above 1000 m.

The longitudinal axis of the monocarps often makes an angle with the stipes that can be as large as 90°. The angle, however, is variable. As it is present in several specimens we do not consider it to be an artefact of pressing and drying.

#### Etymology.

This new species is named in honour of Rocío del Pilar Rojas Gonzales, curator of Herbario Selva Central Oxapampa (HOXA), who collected all but one specimen of this new species.

#### Preliminary IUCN conservation status.

EN B2ab(iii). EOO (56.695 km2) was too large to classify as threatened, but AOO (16 km2) would classify as Endangered. This species is estimated to have 3 locations. All of these lie in heavily deforested areas and deforestation is therefore a major threat to this species. Also, its habitat quality is expected to decline in the near future. No information is available on the current population size and population trend of this species. We assessed this species as Endangered, based on the above information.

#### Other specimens examined.

**PERU**. **Pasco**: Prov. Oxapampa, Distr. Palcazú, Comunidad Nativa Alto Lagarto y 30 de Octubre, 10°09'20"S, 75°25'44"W, 1036 m, 25 Nov 2010, *Rojas & Ortíz 7544* (HOXA, MO, USM, WAG); Parque Nacional Yanachaga-Chemillen, naciente del Río Santa Clara y Lagarto, 10°07'44"S, 75°26'11"W, 1388 m, 5 Aug 2011, *Rojas & Ortíz 7806* (HOXA, MO, USM, WAG); Comunidad Nativa Alto Lagarto, Reserva Comunal Yanesha, 10°08'04"S, 75°22'06"W, 500 m, 13 Nov 2011, *Rojas & Ortíz 8064* (HOXA, MO, USM, WAG); Comunidad Nativa Alto Lagarto, Reserva Comunal Yanesha, 10°08'04"S, 75°22'06"W, 500 m, 10 Feb 2012, *Rojas & Ortíz 8174* (HOXA, MO, USM, WAG); Comunidad Nativa Alto Lagarto, Reserva Comunal Yanesha, 10°08'04"S, 75°22'06"W, 500 m, 30 Oct 2012, *Rojas et al. 8731* (HOXA, MO, USM, WAG); Comunidad Nativa Alto Lagarto-Convento, Reserva Comunal Yanesha, 10°08'04"S, 75°22'06"W, 500 m, 30 Apr 2013, *Rojas & Ortíz 9118* (HOXA, MO, USM, WAG); Comunidad Nativa Alto Lagarto-Convento, Reserva Comunal Yanesha, 10°08'04"S, 75°22'06"W, 500 m, 30 May 2013, *Rojas & Ortíz 9196* (HOXA, MO, USM, WAG); Distr. Villa Rica, Cerro el Ascensor, bosque de protección San Matias-San Carlos, 10°45'28"S, 74°55'92"W 1355 m, 30 Jun 2003, *Perea & Mateo 85* (HOXA).

### Tetrameranthus trichocarpus

Taxon classificationPlantaeMagnolialesAnnonaceae

Maas & Westra sp. nov.

urn:lsid:ipni.org:names:77199060-1

Figs 21 , 22

#### Diagnosis.

*Tetrameranthustrichocarpus* resembles *T.globuliferus* Westra from Ecuador in leaf shape and in the young twigs covered with brown, stellate hairs, but differs by 5-merous (vs. 6-merous) flowers and hairy (vs. glabrous) monocarps, and also by smaller leaves (16–28 vs. 27–37 cm long).

#### Type.

PERU, Loreto: Prov. Maynas, Distr. Medio Putumayo, Inventario Rápido #25, Campamento Bajo Ere, 2°01'07.4"S, 73°15'13.4"W, 125–175 m, 22 Oct 2012, *Ríos et al. 2608* (holotype: F! [F2321026]; isotypes: F!, L!).

#### Description.

*Tree* ca. 10 m tall. Young twigs and petioles densely covered with stiff, brown, mostly stellate hairs to 1–2 mm long. *Leaves*: petioles 8–10 by 3–4 mm; lamina narrowly obovate, 16–28 by 5–8 mm (leaf index 2.5–4), bright shiny green above and pale green below *in vivo*, dark greenish grey above and greenish brown below in *in sicco*, densely covered with brown hairs ≥1 mm long on primary vein and less densely so on secondary veins above, elsewhere rather densely to sparsely covered with stellate and simple hairs, to at last glabrous above, densely to rather densely covered on primary vein and secondary veins below, elsewhere sparsely covered with stellate and simple hairs mainly on lesser veins below, base narrowly acute, apex acuminate (acumen ca. 5 mm long), primary vein slightly prominent to almost flat above, becoming canaliculate *in sicco*, secondary veins 12–20 on either side of primary vein, mostly loop-forming, shortest distance between loops and margin 1–2 mm, tertiary veins percurrent. *Flowers* solitary in axils of leaves; peduncles 7–10 by 2 mm; pedicels 30–35 by 2{-3} mm, to 4 mm diam. in fruit, peduncles and pedicels densely covered with hairs as on twigs; bracts not seen; perianth in 5-merous whorls, petals pale greenish creamy suffused with purple, inner base of inner petals yellowish white, sepals (±) free, narrowly trangular, 4–5 by 10–12 mm, outer side densely covered with hairs as on pedicels to 1 mm long, the inner side same but less densely; outer petals narrowly elliptic-ovate or elliptic-oblong, 30–37 by 10–12 mm, inner side with basal callus to ca. 2/5 of the length and triangular in shape, inner petals narrowly obtriangular-elliptic, about as long as outer petals, slightly narrower than outer petals, markedly recurved about the middle, basal callus on inner side ca. 2/3 of the length and almost touching the side, all petals densely covered with similar though somewhat smaller hairs as on sepals, except for callose parts sparsely so; stamens ∞, apical prolongation of connective shield-like, ca. 1 mm in diam., glabrous; carpels ca. 8, ca. 4 mm long, densely covered with erect hairs to 0.5 mm long on the abaxial side. *Monocarps* 4–6, pinkish green and somewhat shiny *in vivo*, brown and with shriveled wall *in sicco*, ovoid to globose, 4–4.5{-5} by 3–3.5{-4.5} cm, with a conical, obtuse apicule ca. 3–4 mm long, with an oblique constriction (2-seeded forms, only visible *in sicco*), densely to rather densely covered with stiffly, erect, whitish, stellate and simple hairs. *Seeds* (1-)2 per monocarp.

**Figure 21.**
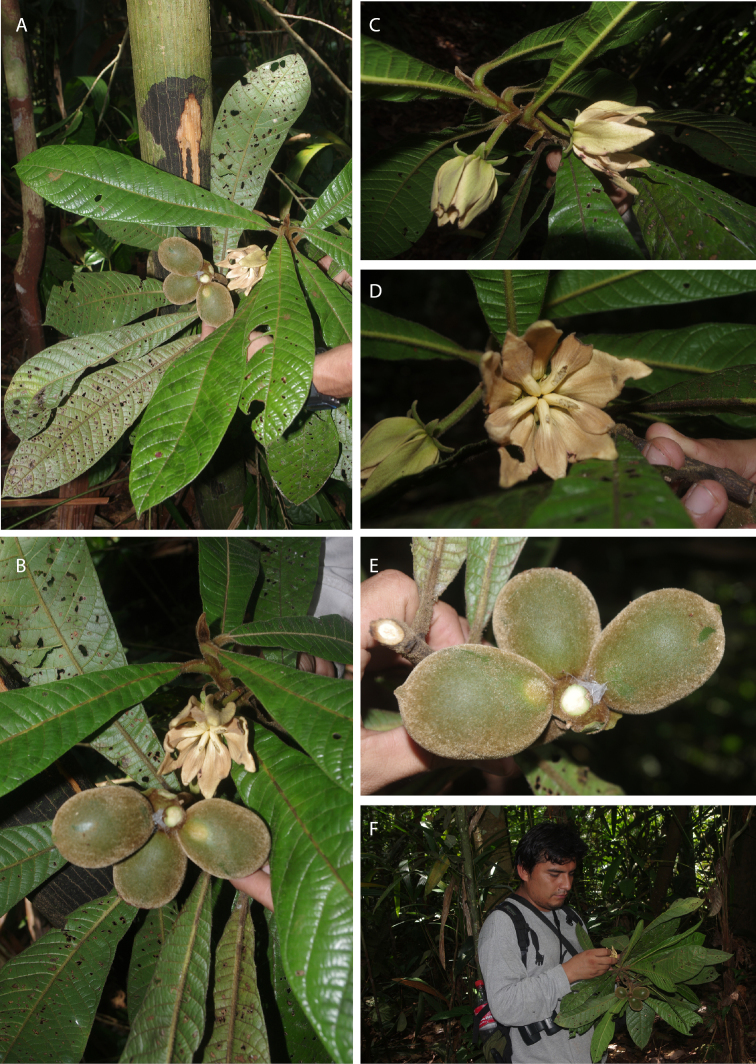
*Tetrameranthustrichocarpus* Maas & Westra. **A** Flowering and fruiting branch **B** detail of same **C** top of branchlet with 2 (young) flowers **D** flower **E** fruit **F** Isau Huamantupa holding collected material. Photographs by I. Huamantupa C.

**Figure 22.**
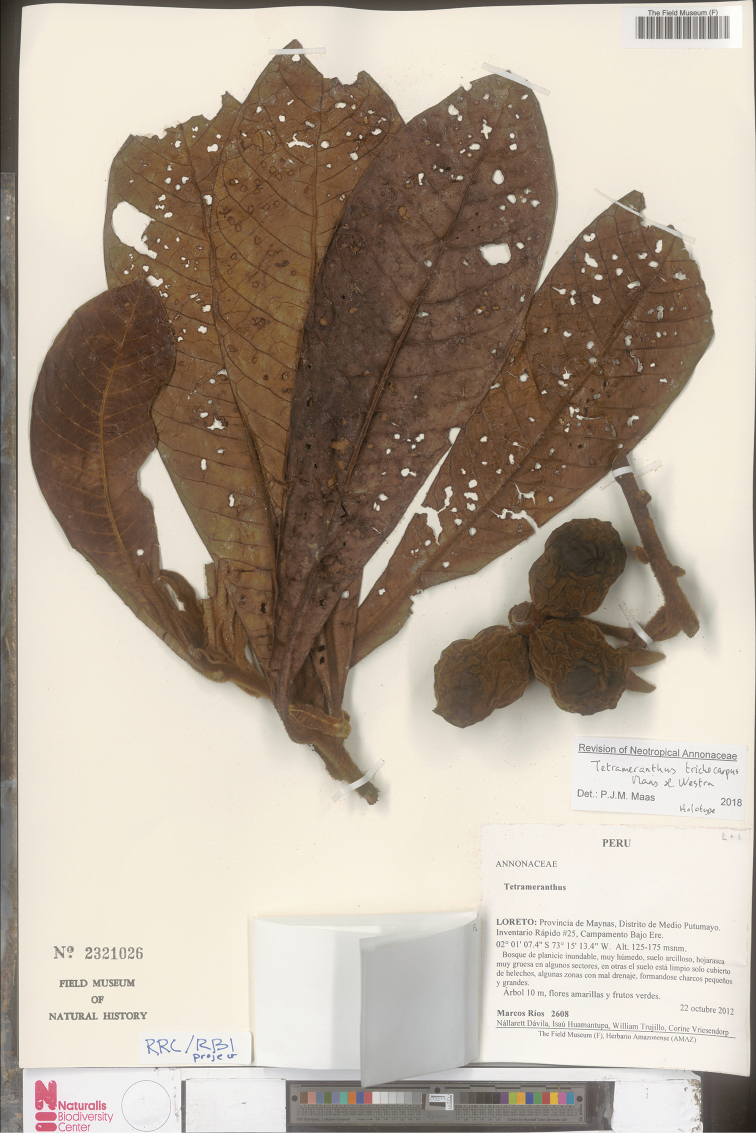
*Tetrameranthustrichocarpus* Maas & Westra. Fruiting branch (*M. Ríos et al. 2608*, holotype F).

#### Distribution.

Peru (Loreto) (Fig. 3).

#### Habitat and ecology.

In moist forest on sandy soil. At an elevation of 125–175 m. Flowering and fruiting: October.

#### Notes.

*Tetrameranthustrichocarpus* is very similar to *T.globuliferus* Westra, from Ecuador (Maas et al. 1988), and also a narrow endemic. Apart from being 5-merous in *T.trichocarpus* vs. 6-merous in *T.globuliferus*, the flowers of the two species resemble each other very much. Both these species share two features with the far-remote *T.guianensis* Westra & Maas, namely a thick fruit wall that shrivels with drying, and an indument of coarse, stellate and simple hairs on vegetative parts. To our knowledge, this is the only species of *Tetrameranthus* with permanently hairy fruits.

#### Preliminary IUCN conservation status.

DD. This species is only known from one collection and therefore no AOO and EOO could be calculated. Also, no other assessment criterium could be used for this species since no information is available on the current population size and population trend of this species. The species seems to occur in a large, pristine forest area and habitat loss does not seem to be an immediate threat to *Tetrameranthustrichocarpus*. However, given the overall lack of data, it was assessed as Data Deficient.

### Xylopia longicaudata

Taxon classificationPlantaeMagnolialesAnnonaceae

Maas & Westra sp. nov.

urn:lsid:ipni.org:names:77199061-1

Fig. 23

#### Diagnosis.

*Xylopialongicaudata* closely resembles *X.uniflora* R.E.Fr. mostly in the leaf size (leaf index 3.5–4.5 for both species), subglabrous stems and young twigs, solitary flowers with basally connate sepals (ca. 2 mm long in *X.longicaudata* and 3–4 mm long in *X.uniflora*), but it differs by the strongly shiny (vs. dull) leaves of which the apex is caudate vs. acuminate, and of which the leaf venation is strongly (vs. hardly) raised.

#### Type.

COLOMBIA, Guainía: Maimachi, Serranía del Naquén, por el camino a Cerro Minas, 02°12'N, 68°13'W, 455 m, 9 Apr 1993, *Madriñan & Barbosa 974* (holotype: NY!; isotype: L!).

#### Description.

*Tree* 15–20 m tall, to ca. 35 cm diam.; young twigs glabrous. *Leaves*: petioles 3–10 by 0.5–1 mm; lamina narrowly elliptic to narrowly obovate, 8–18 by 2–5 cm (leaf index 2.5–3), chartaceous, strongly shiny above *in vivo*, dark brown *in sicco*, pale green below *in vivo*, brown below *in sicco*, glabrous on both sides, base acute, apex caudate (cauda 10–40 mm long), primary vein impressed above, secondary veins hardly countable, ca. 10 on either side of primary vein, strongly raised on both sides, not loop-forming, tertiary veins strongly raised on both sides, strongly reticulate. *Flowers* solitary *in* axils of leaves; pedicels 2–3 mm by 1 mm, sparsely covered with appressed hairs; bracts 4–5, depressed ovate, 1–2 mm long, outer side sparsely covered with appressed hairs; flower buds not seen; sepals basally connate, ovate-triangular, ca. 2 by 3 mm, outer side sparsely covered with appressed hairs; petals creamy yellow *in vivo*, narrowly triangular, 12–13 by 2–3 mm, inner petals narrowly triangular, 7–8 by 1–2 mm, outer side of petals densely covered with appressed, greyish hairs; stamens 1.5–2 mm long, apical prolongation of connective broadly ellipsoid. *Monocarps* and *seeds* not seen, but indicated on the label: “Fruto immaduro verde claro vinosos, maduros externamente rubescente lustroso, internamente, rojo salmon. Semillas negras con arilo basal blanco”.

**Figure 23.**
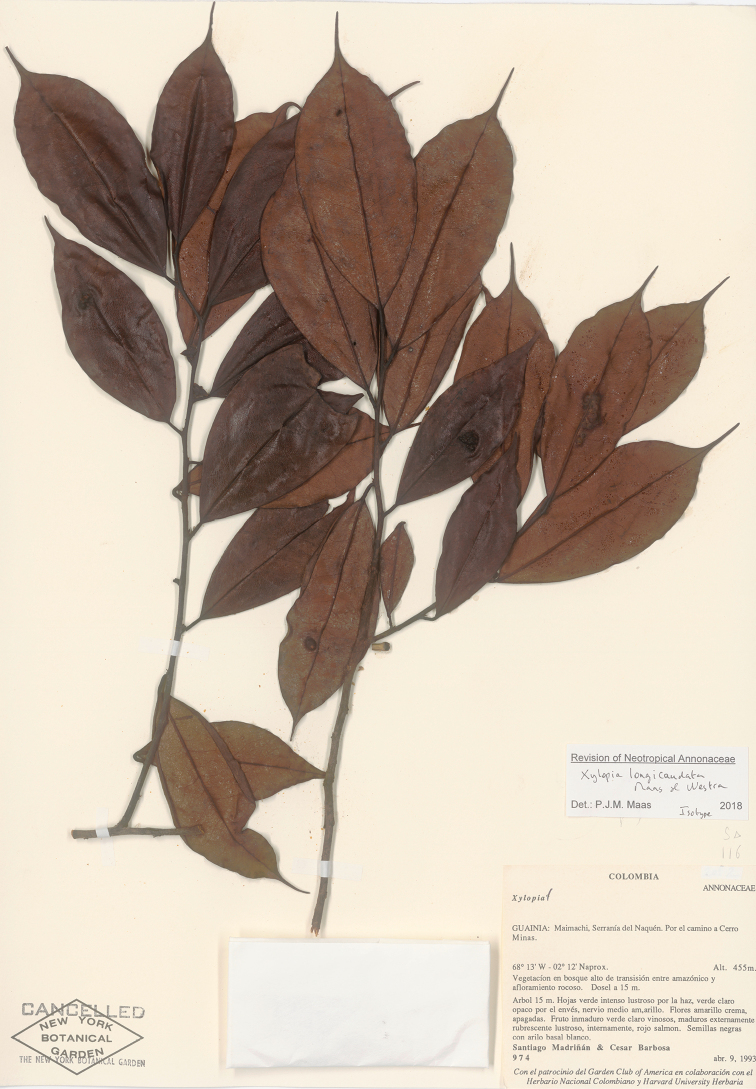
*Xylopialongicaudata* Maas & Westra. (*Madriñan & Barbosa 974*, isotype L).

#### Distribution.

Colombia (Guainía and Vaupés) (Fig. 3).

#### Habitat and ecology.

In high rain forest or high caatinga forest. At elevations of 250–500 m. Flowering and fruiting: April.

#### Notes.

*Xylopialongicaudata* is easily distinguished from other species by the caudate leaf apex. It slightly resembles *X.uniflora* R.E.Fr., described from caatinga forests in Amazonian Brazil (*Ducke RB 29017*, Brazil, Amazonas, Rio Curicuriary, Cajú cataracts, 29 February 1936), but the leaf venation in the latter species is much less raised than in *X.longicaudata* and the apex acuminate instead of caudate.

#### Preliminary IUCN conservation status.

NT. EOO (35.750 km^2^) was too large to classify as threatened, but AOO (20 km^2^) would classify as Endangered. It was determined that this species has 5 locations, none of them in national parks and some in slightly fragmented areas of which the habitat is expected to decline in the near future. Overall, however, this species occurs in large stretches of pristine forest and although no information is available on the current population size and population trend of this species, we expect the population size to be rather large. Therefore this species was classified as Near Threatened.

#### Other specimens examined.

**COLOMBIA**. **Guainía**: “Mitad del camino”, 2°51'127"N, 65°38'339"W, 500 m, 25 Feb 1995, *Córdoba et al. 678* (COL); Sabana Nabuquén, 2°43'188"N, 68°55'312"W, 1 Mar 1995, *Córdoba et al. 769* (COL); Mun. Maimachi, Serranía de Naquen, alrededores del campamento La Planada, 2°12'N, 68°12'W, 320 m, 14 Aug 1992, *Cortés et al. 372* (COL). **Vaupés**: Serranía del Taraira, 10 km al N-W del raudal de la Libertad, 0°58'S, 69°45'W, 250 m, 29 Jul 1993, *Cortés & Rodriguez 646* (COL).

## Supplementary Material

XML Treatment for Annona caput-medusae

XML Treatment for Annona oleifolia

XML Treatment for Guatteria aliciae

XML Treatment for Guatteria attenuata

XML Treatment for Guatteria kamakusensis

XML Treatment for Guatteria pseudoferruginea

XML Treatment for Guatteria pseudorotundata

XML Treatment for Guatteria rotundata

XML Treatment for Guatteria rubiginosa

XML Treatment for Guatteria turrialbana

XML Treatment for Klarobelia rocioae

XML Treatment for Tetrameranthus trichocarpus

XML Treatment for Xylopia longicaudata

## Appendix 1

**Identification list**

Collections are identified by the first collector and number only. The abbreviations behind the collector number refer to the following taxa.

Ann cap = *Annonacaput-medusae* Westra & H.Rainer

Ann ole = *Annonaoleifolia* Westra & H.Rainer

Gua ali = *Guatteriaaliciae* Maas & Erkens

Gua att = *Guatteriaattenuata* Maas & Westra

Gua kam = *Guatteriakamakusensis* Maas & Westra

Gua psf = *Guatteriapseudoferruginea* Maas & Westra

Gua psr = *Guatteriapseudorotundata* Maas & Erkens

Gua rub = *Guatteriarubiginosa* N.Zamora & Maas

Gua tur = *Guaterriaturrialbana* N.Zamora & Erkens

Kla roc = *Klarobeliarocioae* Chatrou

Tet tri = *Tetrameranthustrichocarpus* Maas & Westra

Xyl lon = *Xylopialongicaudata* Maas & Westra

Aldana 10: Gua psf - Alfaro 5445: Gua rub; 5577: Gua rub;

Barclay 3198: Gua psf;

Cabrera R. 1991: Gua psf; 2522: Gua psf – Carrión 517: Gua psr – Cascante 432: Gua tur – Chatrou 417: Ann ole – Córdoba 678: Xyl lon; 769: Xyl lon; 1369: Gua psf – Correa 2307: Ann cap – Correa-Gómez 87: Gua psf; 128: Gua psf; Cortés 372: Xyl lon – Cortés 646: Xyl lon;

De Serdas 588: Gua rub;

Fiaschi 2735: Gua att – Flores 1725: Gua psr - Fonnegra 5935: Ann ole;

Gómez 23305: Gua tur – Grijalva 637: Ann ole – Gudiño 1775: Ann ole;

Herrera 2560: Gua rub; 2701: Gua rub; 3228: Gua rub; 3307: Gua rub; 5249: Gua rub;

Ibañez 5770: Gua ali; 5799: Gua ali; 5813: Gua ali;

Kriebel 2977: Gua tur;

Maas 9516: Gua rub – Madriñan 974: Xyl lon – Monro 4925: Gua rub; 6108: Gua rub;

Perea 85: Kla roc – Pineda 15: Gua psr;

Ríos 2608: Tet tri – Rivera 1693: Gua tur – Rojas 4243: Kla roc; 7544: Kla roc; 7806: Kla roc; 8064: Kla roc; 8174: Kla roc; 8731: Kla roc; 9118: Kla roc; 9196: Kla roc;

Trujillo-C. 3298: Gua psf;

Vargas 1492: Gua tur – Vásquez 22963: Ann ole;

Wurdack 5874: Gua kam;

Zamora 10357: Gua tur; 10363: Gua tur;

## Appendix 2

**Index to scientific names**

*Annona* L.

*caput-medusae* Westra & H.Rainer

*oleifolia* Westra & H.Rainer

*quinduensis* Kunth

*Colpothrinaxaphanopetala* R.Evans

*Guatteria* Ruiz & Pav.

*aliciae* Maas & Erkens

*attenuata* Maas & Westra

*elegantissima* R.E.Fr.

*ferruginea* A.St.-Hil.

*kamakusensis* Maas & Westra

*lucens* Standl.

*modesta* Diels

*pseudoferruginea* Maas & Westra

*pseudorotundata* Maas & Erkens

*rotundata* Maas & Setten

*rubiginosa* N.Zamora & Maas

*schomburgkiana* Mart.

*talamancana* N.Zamora & Maas

*turrialbana* N.Zamora & Erkens

sp. 2 Maas & Westra

*Klarobelia* Chatrou

*napoensis* Chatrou

*pandoensis* Chatrou

*peruviana* (R.E.Fr.) Chatrou

*pumila* Chatrou

*rocioae* Chatrou

*Raimondia* Saff.

*Rollinia* A.St.-Hil.

*Tetrameranthus* R.E.Fr.

*globuliferus* Westra

*guianensis* Westra & Maas

*trichocarpus* Maas & Westra

*Xylopia* L.

*longicaudata* Maas & Westra

*uniflora* R.E.Fr.
